# The Profile and Dynamics of RNA Modifications in Animals

**DOI:** 10.1002/cbic.201700093

**Published:** 2017-04-27

**Authors:** Pieter van Delft, Alper Akay, Sabrina M. Huber, Christoph Bueschl, Konrad L. M. Rudolph, Tomás Di Domenico, Rainer Schuhmacher, Eric A. Miska, Shankar Balasubramanian

**Affiliations:** ^1^ Department of Chemistry University of Cambridge Lensfield Road Cambridge CB2 1EW UK; ^2^ Gurdon Institute University of Cambridge Tennis Court Road Cambridge CB2 1QN UK; ^3^ Department of Genetics University of Cambridge Downing Street Cambridge CB2 3EH UK; ^4^ Center for Analytical Chemistry Department of Agrobiotechnology University of Natural Resources and Life Sciences Vienna Konrad-Lorenz-Strasse 20 3430 Tulln an der Donau Austria; ^5^ Wellcome Trust Sanger Institute Wellcome Trust Genome Campus Cambridge CB10 1SA UK; ^6^ Cancer Research UK Cambridge Institute University of Cambridge Robinson Way Cambridge CB2 0RE UK

**Keywords:** *Caenorhabditis elegans*, isotopic labeling, mass spectrometry, RNA modifications, stress response, tRNA

## Abstract

More than a hundred distinct modified nucleosides have been identified in RNA, but little is known about their distribution across different organisms, their dynamic nature and their response to cellular and environmental stress. Mass‐spectrometry‐based methods have been at the forefront of identifying and quantifying modified nucleosides. However, they often require synthetic reference standards, which do not exist in the case of many modified nucleosides, and this therefore impedes their analysis. Here we use a metabolic labelling approach to achieve rapid generation of bio‐isotopologues of the complete *Caenorhabditis elegans* transcriptome and its modifications and use them as reference standards to characterise the RNA modification profile in this multicellular organism through an untargeted liquid‐chromatography tandem high‐resolution mass spectrometry (LC‐HRMS) approach. We furthermore show that several of these RNA modifications have a dynamic response to environmental stress and that, in particular, changes in the tRNA wobble base modification 5‐methoxycarbonylmethyl‐2‐thiouridine (mcm^5^s^2^U) lead to codon‐biased gene‐expression changes in starved animals.

Canonical nucleobases, especially those of ribonucleic acids, are naturally subject to diverse modification. The number of identified modified ribonucleosides has grown to over a hundred, and more than half of them can be found in eukaryotic RNA.^1^ Although they have been described in ribosomal RNAs (rRNAs), messenger RNAs (mRNAs) and various noncoding RNA species, the highest frequency and greatest chemical diversity of modified nucleosides can be found in transfer RNAs (tRNAs).^2^ There, their presence contributes to the correct functioning of the protein synthesis machinery by providing stability, structure and adding diversity in molecular recognition.^2^, ^3^ In the cases of bacteria and yeast, almost a complete picture of genes and pathways leading to tRNA modifications exists and many homologous proteins have been described in other, higher eukaryotes.^4^

The recent development of sequencing‐based detection methods has facilitated the identification of modifications in mRNAs. Examples include 5‐methylcytidine (m^5^C), pseudouridine (ψ), *N* ^1^‐methyladenosine (m^1^A) and *N* ^6^‐methyladenosine (m^6^A).^5^ The last of these was recently identified as the first reversible RNA modification and provided direct evidence of the existence of dynamic nucleoside modification processes.^6^

The idea of RNA modifications as part of a dynamic process is not completely new or limited to mRNA, and a few observations have collectively implicated a dynamic mechanistic role for RNA modifications. They include the presence of tissuedependent levels of modifications, the existence of variable methylation of specific ribosomal base residues, links between specific tRNA modifications and disease and progressive RNA modification throughout neural cell ageing.^7^ Furthermore, RNA modifications have been linked to stress response in multiple organisms.^8^, ^9^ It is thus becoming more evident that temporal and spatial control of RNA modifications might be ubiquitous and important for correct functioning of the RNA. The majority of studies on the dynamics of modified ribonucleosides characterise single or a few modifications at a time due to technical limitations. Most sequencing‐based methods rely either on antibodies that can recognise certain modifications or on reverse transcription errors that can be interpreted for the presence of modified nucleosides, both of which limit the number of RNA modifications that can be analysed simultaneously. Mass‐spectrometry‐based methods require chemically synthesised nucleosides that can be used as reference standards during the measurements to establish the presence and identity of any given modification. This limits the analysis to modifications for which reference standards are readily available. There are a few studies that describe MS‐based methods that overcome the need for synthetic references and enable the characterisation of multiple nucleoside modifications at the same time.^10^, ^11^ In vivo, RNA modification landscapes of animals and their dynamic nature under different stress conditions are still unknown. Here we describe a method that enables the untargeted and automated characterisation of the most abundant RNA modifications in a multicellular organism under multiple stress conditions.

In this approach, we use metabolic labelling to generate bio‐isotopologues of the *Caenorhabditis elegans* transcriptome and apply them as reference standards in a HRMS‐based method for the identification of RNA modifications and their dynamic changes upon heat shock and starvation. Our results show that several RNA modifications exhibit changes in abundance levels upon stress induction, in a reversible manner. In particular, changes in the tRNA wobble base modification 5‐methoxycarbonylmethyl‐2‐thiouridine (mcm^5^s^2^U) lead to codon‐biased gene‐expression changes in starved animals. We based our method on the powerful software tool MetExtract, originally developed for the automated extraction of metabolite‐derived LC‐MS signals from LC‐HRMS full‐scan data obtained from isotopically labelled biological samples.^12^ We reasoned that this software could be adapted for the untargeted identification of nucleoside modifications in RNA from isotopically labelled whole organisms such as *C. elegans*. d‐[^13^C_6_]Glucose had previously been used to isotopically label the bacterial transcriptome as it feeds into the pentose phosphate pathway (Figure S1 A in the Supporting Information).^10^ We set out to culture *C. elegans* in the presence of isotopically labelled bacteria, serving as food, to transfer the isotopes from the *Escherichia coli* transcriptome to the *C. elegans* transcriptome and to generate bio‐isotopologues of every modified ribonucleoside present in the animal. We first grew HT115 *E. coli* in the presence of d‐[^13^C_6_]glucose by adaption of previously reported methods.^10^ In particular, we additionally added the amino acids from Dulbecco's modified Eagle's medium (DMEM) to the culture medium to avoid ^13^C incorporation into amino acids involved in de novo nucleotide synthesis and thus to achieve more uniform ^13^C_6_‐labelling rather than ^13^C_*n*_‐labelling (Figure S1 B, C).

We then cultured *C. elegans* in the presence of the labelled bacteria for three generations, after which the nematodes were harvested for total RNA isolation (Figure 1). The degree of success of isotope incorporation into the *C. elegans* transcriptome was then assessed by LC‐MS/HRMS analysis of enzymatically digested total RNA samples and subsequent assessment of the high‐resolution mass spectrum of adenosine (Figure S1 C). Having established the ^13^C‐labelling of *C. elegans* RNA, we aimed to determine its most abundant RNA modifications. We thus fractionated total RNA into small (<200 nt) and large (>200 nt) RNAs (Figure S2) to increase the sensitivity for the less abundant—primarily tRNA—modifications in the small‐RNA fraction.

**Figure 1:**
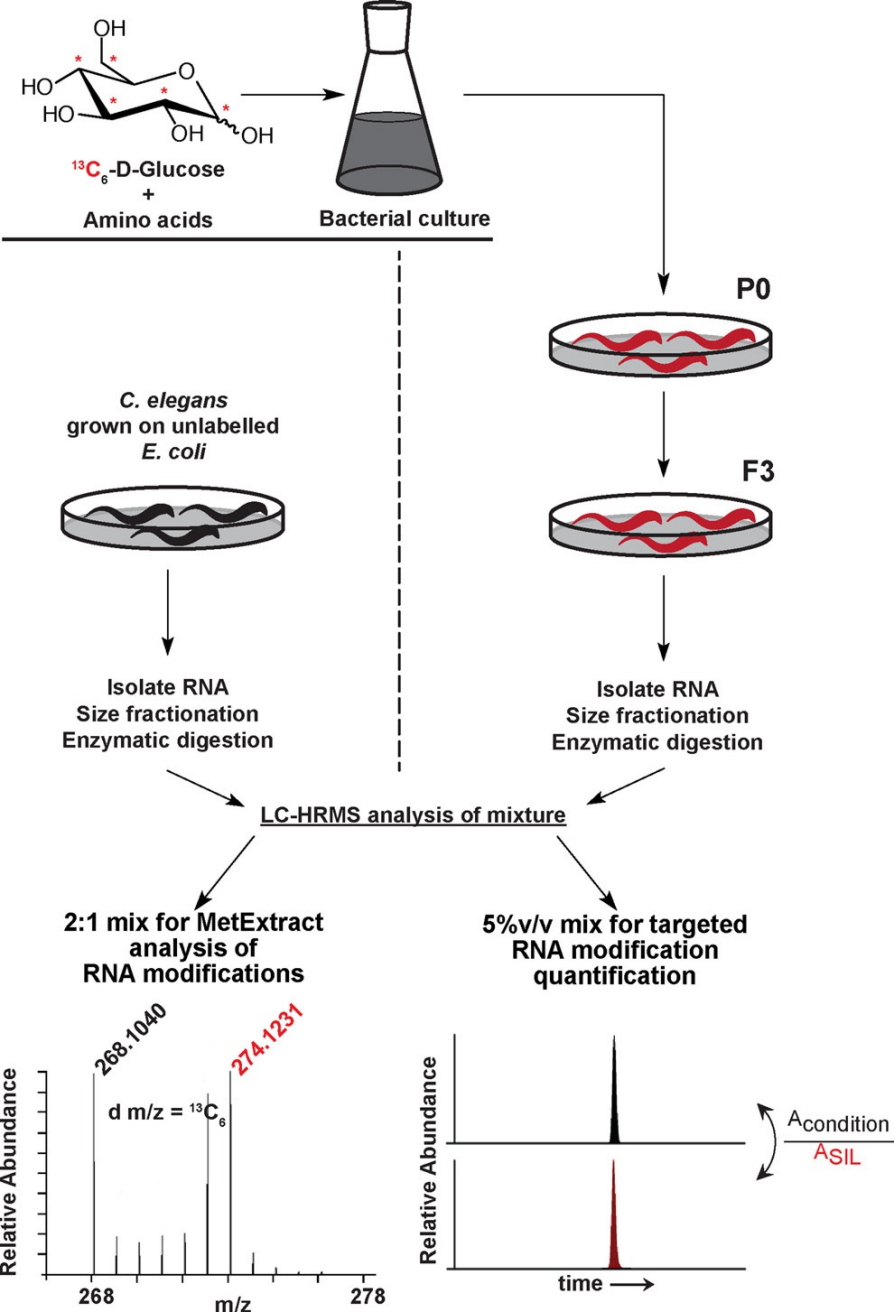
^13^C‐Labelling of the *C. elegans* transcriptome. *C. elegans* larvae were fed for three generations either with heavy‐labelled *E. coli* that had been grown in media containing d‐[^13^C_6_]glucose and amino acids, or with unlabelled *E. coli*. Total RNA from labelled or unlabelled animals was isolated, size fractionated and subjected to LC‐MS/HRMS analysis. A 1:2 ratio of RNA from unlabelled and labelled animals was used with MetExtract analysis for algorithm‐based determination of RNA modifications. A 5 % (*v*/*v*) mix of labelled RNA in unlabelled RNA was used for relative quantification of RNA modifications.

A 2:1 mixture of labelled and unlabelled RNA (for each fraction) was then measured by HPLC‐HRMS in the 250–500 *m*/*z* range (Figure 1). Unlabelled RNA was obtained from control nematodes grown under standard conditions with unlabelled bacteria as a food source. The recorded data were then analysed by using a nucleoside‐adapted version of the MetExtract software. The raw LC‐HRMS data were mined for all ions co‐eluting and showing a mass shift corresponding to native and ^13^C_6_‐labelled isotopologues to produce an *m*/*z* list containing high‐resolution masses likely to correspond to the different RNA nucleosides present in the mixture and readily detectable. These masses were then manually screened against masses from known RNA modifications listed in RNA modification databases to obtain potential structures.^1^

Thus, at this stage we had identified RNA modifications by their accurate masses and those of their corresponding, co‐eluting, ^13^C_6_ isotopologues. (Complete structural assignment was later achieved as described in the next section.) For this reason some residues were found more than once: the mass of methylcytidine, for instance, was found three times, presumably m^5^C, 3‐methylcytidine (m^3^C) and 2′‐*O*‐methylcytidine (Cm). Altogether, we identified 21 and 26 modifications in the *C. elegans* large‐ and small‐RNA fractions, respectively (Table 1). In the small‐RNA fraction we observed the *m*/*z* value corresponding to 3‐(3‐amino‐3‐carboxypropyl)uridine (acp^3^U) and its corresponding ^13^C_6_ isotopologue, a modification that has not been previously described in eukaryotic RNA, thus demonstrating the potential of our approach for the discovery of modified ribonucleosides in a completely untargeted fashion.

**Table 1:** List of all modifications that could be assigned to known ribonucleoside modifications from a list of high‐resolution masses obtained from the MetExtract algorithm.

Modified residue	No. found in RNAs		Abbrev.
	<200 nt	>200 nt	
methylcytidine	3	3	mC*
methyluridine/methyl‐pseudouridine	2	3	mU*/mψ
*N* ^4^‐acetylcytidine	1	1	ac^4^C
methyladenosine	3	3	mA*
5‐methylaminomethyluridine	1	1	mnm^5^U
dimethyladenosine	1	1	m_2_A*
methylguanosine	3	3	mG*
dimethylguanosine	1	2	m_2_G*
trimethylguanosine	1	1	m_3_G*
5‐methoxycarbonylmethyl‐2‐thiouridine	1	1	mcm^5^s^2^U
*N* ^6^‐isopentenyladenosine	1	1	i^6^A
*N* ^6^‐threonylcarbamoyl‐adenosine	1	1	t^6^A
inosine	1		I
methylinosine	1		mI*
5‐methylaminomethyluridine	1		mnm^5^U
5‐(Carboxy(hydroxy)methyl)uridine	1		mchm^5^U
methyl ester			
queuosine	1		Q
2‐methylthio‐*N* ^6^‐threonyl‐carbamoyl	1		ms^2^t^6^A
adenosine			
3‐(3‐amino‐3‐carboxypropyl)uridine	1		acp^3^U

A full list of extracted high‐resolution masses is provided in Table S1. Superscripted numbers indicate the positions of the modifications on the nucleobases. *: Modified ribonucleoside residues detected with known number and kind of substituents but unknown position(s) of modification(s) due to their identification by high‐resolution mass *only*.

Next, we aimed to quantify the RNA modification landscape of *C. elegans* under physiological stress to improve understanding of the relationship between modified RNAs and stress response pathways. Heat stress and starvation are two stress conditions that lead to large‐scale gene‐expression changes in animals.^13^ We either subjected *C. elegans* larvae to heat shock by shifting adult animals from 20 to 37 °C for 4 h or we starved the animals by removing their food source for 4 h at 20 °C. Within 4 h either of heat stress or of starvation, adult *C. elegans* animals showed gene‐expression changes that were specific to each stress condition: upon heat stress several heat‐shock factors were strongly upregulated (Figure S4 A), whereas geneexpression changes upon starvation strongly overlapped with previous starvation data on *C. elegans* (Figure S4 B).

Having established the stress conditions, we then aimed to quantify the changes in RNA modifications under these conditions of heat shock and starvation. We focused on RNA modifications that we had profiled earlier (Table 1) and could easily detect. By using the HPLC‐MS/HRMS comparative quantitation method, we tried to capture the changes in the RNA modification landscape of *C. elegans* before and after stress induction. This was achieved by spiking in digested ^13^C_6_‐labelled RNA at 5 %, *v*/*v* into unlabelled digested RNA obtained from stressed (heat‐shocked or starved) or control animals and subjecting the mixture to targeted LC‐MS/HRMS analysis. Where possible, standards from commercial and synthetic sources were used to identify the presence of most residues unequivocally (m^1^A, m^6^A, i^6^A, *N* ^4^‐acetylcytidine (ac^4^C), m^5^C, Cm, Gm, *N* ^2^,*N* ^2^‐dimethylguanosine (m^2^_2_G), 7‐methylguanosine (m^7^G), I, Um, 5‐methyluridine (m^5^U), mcm^5^s^2^U); moreover, with the added selectivity of tandem mass spectrometry (MS/HRMS), other residues for which standards are not available could also be assigned by taking the co‐elution of their fragments masses into account (i.e., t^6^A, ms^2^t^6^A, acp^3^U). We were unable to resolve putative m^1^G and m^2^G. However, because these residues do not change we did not proceed further in structurally identifying them and refer to these residues as mG (base‐methylated guanosine). The peak areas of each modified nucleoside and its corresponding ^13^C_6_ bio‐isotopologues, present in the spike‐in, were then determined in each sample, and their ratio was calculated (A_condition_/A_SIL_, SIL=stable isotope labelling) to obtain a normalised peak area for every modification under three different sets of conditions (control, starved, heat shocked, Figure 1).

Next, we compared the normalised peak areas of stressexposed nematodes with those of control animals for every modification (Figure 2 A, B). We observed only two significant (*p*<0.05) RNA modification changes in heat‐shocked animals: the *N* ^2^,*N* ^2^,7‐trimethylguanosine (m^2,2,7^G) residue showed a strong reduction in the large‐RNA fraction, and mcm^5^s^2^U levels showed a modest decrease in the small‐RNA fraction. On the other hand, starvation induced numerous RNA modification changes in both the large‐ and the small‐RNA populations. Interestingly, in the large‐RNA fraction in particular, we observed that several base methylations increased upon starvation; these included m^5^C, m^1^A, m^7^G, m^5^U, m^2,2,7^G and m^2^_2_G. Observable RNA modification changes upon starvation in the small‐RNA fraction were constrained to two known tRNA wobble base modifications: ac^4^C and mcm^5^s^2^U levels both decreased in starved animals. We validated our results by absolute quantification of two of the RNA modifications by a method previously reported by us^14^ in which m^5^C levels in the large‐RNA fraction were indeed found to be significantly increased upon starvation (Figure S5 A, *p*<0.05), whereas m^6^A levels in the large‐RNA fraction showed no change between control and starved animals (Figure S5 B). Both these absolute quantitations are in line with the relative quantification method based on bio‐isotopologues presented here. Overall, this allowed us to determine the relative abundance of multiple RNA modifications in whole animals upon stress induction.

**Figure 2:**
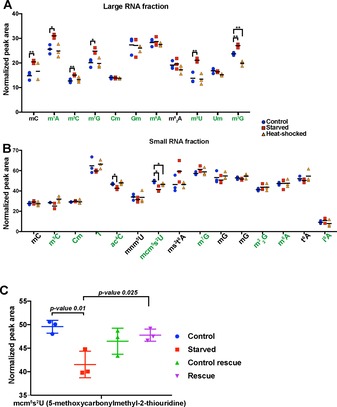
Relative quantification of RNA modifications in A) large‐RNA or B) small‐RNA fractions variously from control (blue), starved (red) or heat‐shocked (yellow) *C. elegans* [* *p*<0.05, ** *p*(adjusted)<0.05]. The modifications in green were identified with the aid of the added selectivity of MS/HRMS and a reference standard from a synthetic or commercial source. C) Normalised ratios of mcm^5^s^2^U in small‐RNA fraction upon starvation and after starvation rescue (to avoid age‐dependent changes, age‐matched control animals were used alongside the rescue animals).

To gain a full picture of the dynamic nature of RNA modifications, we tried to determine whether or not the stress‐induced changes in RNA modifications are reversible. To this end we rescued starved animals by reintroducing food and compared the changes in RNA modifications to those in age‐matched control animals. All RNA modifications that were significantly altered upon starvation showed a general trend of reversal upon starvation rescue, with, for example, m^5^C and m^5^U in the large‐RNA fraction (Figure S6) and mcm^5^s^2^U in the small‐RNA fraction reversing significantly. As shown in detail for mcm^5^s^2^U in Figure 2 C the levels of this modification were significantly lowered upon starvation, whereas upon rescue (*p* value <0.01) its levels no longer differed from those in the control population. Absolute quantification of m^5^C and m^6^A in the large‐RNA fractions of rescued animals and control animals (Figure S5 C, D) were again in line with our relative quantification measurements: that is, we measured no difference between control and rescued populations for these modifications. Our results show that several RNA modifications not only respond to stress conditions, but are also dynamically regulated between stress and normal growth conditions of *C. elegans*. We were particularly interested in the tRNA wobble base modification mcm^5^s^2^U, which showed levels that were significantly reduced in starved animals but were then restored to wild‐type levels upon reintroduction of food. mcm^5^s^2^U is found in tRNA‐Lys^UUU^, tRNA‐Glu^UUC^ and tRNA‐Gln^UUG^ at position U_34_ of the anticodon loop and it is required for cell viability and animal development.^15^ Loss of mcm^5^s^2^U has been linked to inefficient translation, ribosome stalling and protein misfolding in cases of genes that are enriched for codons AAA, GAA and CAA.^9^

To test whether starvation‐induced reduction of mcm^5^s^2^U levels leads to codon‐specific changes in gene expression, we analysed the codon enrichment of AAA, GAA and CAA in comparison with all other codons, among genes that show differential expression upon starvation (Figure S4). Surprisingly, the codons AAA and GAA show significant enrichment in differentially expressed genes after starvation (Figure 3 A). Previously, such codon effects on translation efficiency were observed upon performing either ribosomal profiling or proteomics experiments.^9^ Our results show that such codon‐biased effects of tRNA modifications can be captured by sequencing RNA, most likely due to stabilisation of these mRNAs on ribosomes.9a We did not see any such codon enrichment among the differentially expressed genes in heat‐shocked animals (Figure 3 B), thus indicating that the enrichment of the codons AAA and GAA is specific to starvation response.

**Figure 3:**
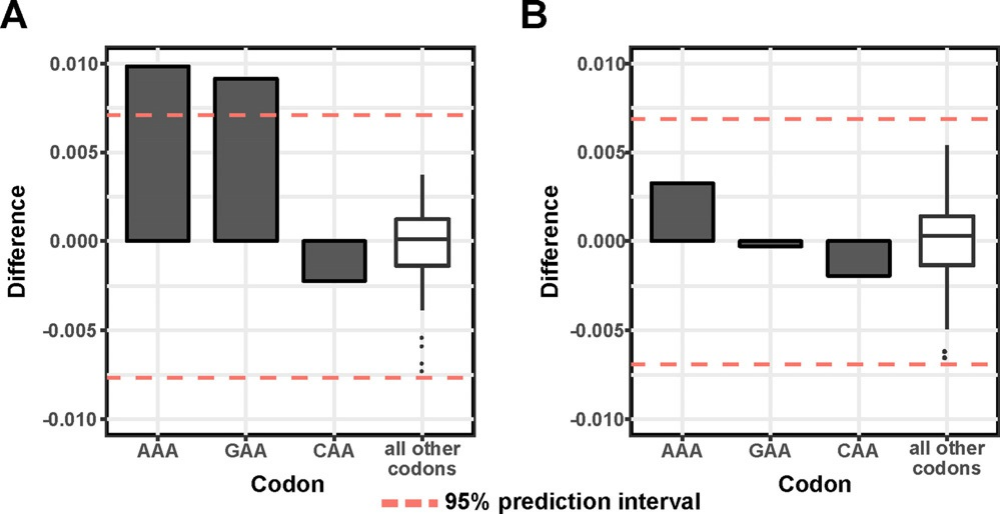
Starvation‐induced codon enrichment among differentially expressed genes. Codon enrichment of AAA, GAA and CAA in comparison with all other codons in A) starved animals, and B) heat‐shocked animals (95 % prediction intervals are marked by dashed lines, difference is calculated by weighing codon abundance to gene expression levels in treated vs. control samples).

In summary, we used stable isotopes to label the transcriptome of the multicellular model organism *C. elegans*. We were able to identify the most abundant and readily detectable modifications in the large‐ and small‐RNA fractions in an automated and untargeted fashion by using the labelled RNA in LC‐MS/HRMS experiments. Furthermore, we used these bioisotopologues for relative LC‐MS/HRMS quantification to show that some of these modifications exhibit dynamic changes in their global levels as a result of heat shock or dietary restriction. By combining RNA modification measurements with transcriptome analysis, we show that the wobble base modification mcm^5^s^2^U levels correlate with codon‐biased gene expression. Currently, it is not easy to distinguish changes in RNA modification levels from changes in RNA levels where a certain modification exists. Nevertheless, by using bio‐isotopologues to measure RNA modification levels and combining this with transcriptome‐wide analysis of gene expression it is possible to uncover important links between RNA modifications and animal physiology. It will be important to explore the mechanisms of how RNA base modifications respond to environmental stress in eukaryotes in future work.

## Experimental Section

Starter cultures of *E. coli* HT115 strain were grown overnight in M9 minimal medium supplemented with d‐glucose (^12^C or ^13^C, 0.2 %, *w*/*v*) and MgSO_4_ (1 mm). Starter cultures were used for growing fresh *E. coli* HT115 cultures in M9 minimal medium to an OD_600_ of 0.8–1.0. Bacterial cultures were pelleted for RNA isolation or *C. elegans* culture. Resuspended bacteria were seeded to NGM‐N agarose plates, and the plates were left to dry overnight. Ten larval stage 1 animals were placed on seeded NGM‐N agarose plates with the labelled bacteria and left to grow for two generations. Adult F1‐generation animals were bleached to obtain a synchronous population of F2‐generation animals. F2 animals were placed on freshly seeded NGM‐N plates with labelled bacteria and grown to adult stage. Adult animals were washed off the plates and pelleted before DNA and RNA isolation. Synchronised populations of L1 animals were grown until the young adult stage. For heat stress, animals were transferred to 37 °C incubators for 4 h with food. After 4 h, animals were washed off the plates, cleaned from bacteria by washing in M9 buffer (3×) and stored in TRIsure *(Bioline*) reagent for subsequent RNA isolation. For starvation experiments, young adult stage animals were washed off the plates using M9 buffer and plated either on food plates for control or on empty plates for starvation. Animals were left at 20 °C for 4 h. After 4 h, animals were washed off the plates, cleaned by washing in M9 buffer (3×) and stored in TRIsure reagent for RNA isolation. For rescue experiments, control and starved animals were plated on plates with food and left for 8 h at 20 °C.

Isolated total RNA (see the Supporting Information) was fractionated into “small” and “large” RNAs with the aid of a Quick‐RNA MiniPrep kit (*Zymo Research*) according to the manufacturer's instructions.

Enzymatic digests of size‐fractionated RNA were mixed in a 1:2 ratio (m/m based on RNA amounts). For the <200 nt fraction the unlabelled RNA (1.5 μg) was added to the ^13^C SIL small RNA (3 μg). For the >200 nt fractions, 3 and 6 μg, respectively, were used. LC‐HRMS was performed with a Thermo Ultimate 3000 UHPLC system equipped with a Waters HSS‐T3 column and coupled to a Thermo Qexactive hybrid mass spectrometer. LC conditions were as follows: H_2_O/MeCN solvent system (formic acid, 0.1 %); HRMS was performed in Full MS‐SIM mode, resolution 35 000, scan range 250–500 *m*/*z*.

Using the absolute concentration of rC as a reference for the amount of RNA in each sample (Table S2) we adjusted the samples' RNA concentrations. ^13^C‐Labelled RNA digest (5 %, *v*/*v*) was added as the internal reference standard. With the same experimental setup, LC‐MS/HRMS was then performed with the machine operated in MRM mode; resolution 35 000 and inclusions as listed in Table S3.

Complete experimental procedures are provided in the Supporting Information.

## Conflict of interest

*The authors declare no conflict of interest*.

## Supporting information

As a service to our authors and readers, this journal provides supporting information supplied by the authors. Such materials are peer reviewed and may be re‐organized for online delivery, but are not copy‐edited or typeset. Technical support issues arising from supporting information (other than missing files) should be addressed to the authors.

Supplementary
