# Supplementary material for: The Profile and Dynamics of RNA Modifications in Animals
Source: Chembiochem. 2017 Apr 27;18(11):979–84. doi: 10.1002/cbic.201700093 (PMC5784800; doi:10.1002/cbic.201700093)
Supplement: Supplementary file 1 — Supplementary [file CBIC-18-979-s001.pdf]

CHEMBIOCHEM

Supporting Information

The Profile and Dynamics of RNA Modifications in Animals

Pieter van Delft+,[a] Alper Akay+,[b, c] Sabrina M. Huber+,[a] Christoph Bueschl,[d]
Konrad L. M. Rudolph,[b, c, e] Tomás Di Domenico,[b, c, e] Rainer Schuhmacher,[d]
Eric A. Miska,*[b, c, e] and Shankar Balasubramanian*[a, f]

cbic_201700093_sm_miscellaneous_information.pdf

Author Contributions

The manuscript was written through contributions of all authors.

LC-HRMS for MetExtract analysis........ 2
MetExtract data processing of LC-HRMS full scan data. 2
Quantitative LC-MS/MS analysis to determine sample rC concentration. 3
Comparative quantitation of RNA nucleoside base modifications........ 3
Quantitative LC-MS/MS analysis of m^5C and m^6A........ 4
C. elegans husbandry 4
Culturing *E. coli* on ^{12}C or ^{13}C D-glucose 4
^{13}C labeling of *C. elegans* using ^{13}C labeled *E. coli* as food source........ 5
Heat stress and starvation of *C. elegans* larvae 5
C. elegans disruption for total RNA isolation........ 5
Total RNA isolation........ 6
Small/large RNA fractionation 6
RNA digestion for LC-MS/MS analysis 6
Analysis of codon usage 7
Figure S1: ^{13}C-labelling of the *C. elegans* transcriptome. 8
Figure S2: Agilent TapeStation electropherograms of large and small fractions. 9
Figure S3: Calibration curve used for the quantification of rC........ 10
Figure S4: RNA-Seq data analysis of heat-shocked and starved animals........ 10
Figure S5: Absolute quantification of RNA modifications........ 11
Figure S6: Dynamic changes of RNA modifications in *C. elegans*. 12
References 13
SI TABLES 1-4........14

LC-HRMS for MetExtract analysis

50 µl enzymatic digests of size fractionated RNA were mixed in a 1 : 2 ratio (m/m based on RNA amounts). For the < 200 nt fraction 1.5 µg of the unlabeled RNA was added to 3 µg of the ^{13}C SIL small RNA with a final volume of 75 µl. For the > 200 nt fractions, 3 and 6 µg were used respectively (V_{tot} = 75 µl). 30 µl injections of the nucleoside mixtures were then analyzed using a Thermo Ultimate 3000 UHPLC system equipped with a Waters HSS-T3 column (2.1 x 100 mm, 1.8 µm particle size) and coupled to a Thermo Qexactive hybrid mass spectrometer. LC conditions were as follows: H_2O – MeCN solvent system containing 0.1 % formic acid; 0 – 100 % multistep gradient over 8 minutes. HRMS was performed using a HESI source (4 kV, capillary T: 350 ^{o}C, sheath gas: 30, auxiliary gas: 2, heater T: 350 ^{o}C. The machine was set in Full MS-SIM mode, resolution: 35,000, scan range 250 – 500 m/z.

MetExtract data processing of LC-HRMS full scan data

Raw-LC-HRMS data files were converted to mzXML format with ProteoWizard (v. 3.0.5274, http://proteowizard.sourceforge.net/, centroiding algorithm, *Thermo Fisher Scientific*). Pairs of ion signals from native and corresponding ^{13}C labeled compounds containing 5 or 6 ^{13}C atoms were searched for in each recorded MS scan (Δ *m/z* between ^{12}C and ^{13}C 1.00335 amu, maximum allowed *m/z* deviation 3 ppm, minimum signal intensity 20,000 counts) and subsequently clustered into signal bins (maximum allowed *m/z* deviation per bin 8 ppm). For each signal bin, the EICs of the native and corresponding ^{13}C-labeled ions were extracted and inspected for co-eluting chromatographic peak pairs representing the native and the ^{13}C-labeled forms of the same metabolite (EIC width ± 5 ppm, minimum Pearson correlation of co-eluting chromatographic peaks 0.85). Detected chromatographic peak pairs originating from different adduct or in-source fragment ions of the same compound were then convoluted into groups each group representing a compound of interest (minimum Pearson correlation 0.85) (SI_TABLE 1).

Quantitative LC-MS/MS analysis to determine sample rC concentration

Prior to the SIL based comparative quantitation of nucleoside base modifications, the absolute amount of cytosine in digests of size fractionated *C. elegans* RNA samples of control, heat shock, dietary restricted and rescued samples was determined using SIL addition quantitative LC-MS/MS analysis using a method previously reported by us.[1] The RNA digests (4 – 10 µg in 50 µl) were diluted 20x and a 100 nM solution of [$^{15}N_2{}^{13}C$]-rC was added (25 nM final concentration). A dilution series of rC was then prepared in the range of 0.75 – 11,250 nM containing 25 nM [$^{15}N_2{}^{13}C$]-rC in every calibration point.

Calibration standards and samples were measured at 10 µl injections using a Thermo Ultimate 3000 UHPLC system equipped with a Waters HSS-T3 column (2.1 x 100 mm, 1.8 µm particle size) and coupled to a Thermo Qexactive hybrid mass spectrometer. LC conditions were as follows: H_2O – MeCN solvent system containing 0.1 % formic acid; 0 – 100 % multistep gradient over 5 minutes (0 – 30 % 3 min; 100 % 4 min; 100 % 5 min) and 1 minute column regeneration time. LC-MS/HRMS was performed using a HESI source (4 kV, capillary T: 350 ^{o}C, sheath gas: 30, auxiliary gas: 2, heater T: 350 ^{o}C. The machine was operated in MRM mode; resolution: 35,000, isolation window: 3.0 m/z, NCE: 10 and the following inclusions: rC m/z: 244 and [$^{15}N_2{}^{13}C$]-rC m/z: 247. Data was processed using Thermo XCalibur 2.2.44 Quanbrowser (SI_TABLE 2).

Comparative quantitation of RNA nucleoside base modifications

Using the absolute concentration of rC as a reference for the amount of RNA in each sample we adjusted the samples' RNA concentration to the lowest amount for both the < 200 nt (8.8 µM) and > 200 nt fraction (16 µM) in 57 µl. Next, 3 µl (5 v/v %) of ^{13}C labeled RNA digest (30 µg in 100 µl) was added as the internal reference standards.

LC-MS/MS was then performed using a Thermo Ultimate 3000 UHPLC system equipped with a Waters HSS-T3 column (2.1 x 100 mm, 1.8 µm particle size) and coupled to a Thermo Qexactive hybrid mass spectrometer. LC conditions were as follows: H_2O – MeCN solvent system containing 0.1 % formic acid; 0 – 100 % multistep gradient over 5 minutes (0 – 30 % 3 min; 100 % 4 min; 100 % 5 min) followed by 1 minute of column regeneration time. LC-MS/HRMS was performed using a HESI source (4 kV, capillary T: 350 ^{o}C, sheath gas: 30, auxiliary gas: 2, heater T: 350 ^{o}C. The machine was operated in MRM mode; resolution: 35,000, isolation window: 3.0 m/z, NCE: 10 and inclusions as listed in SI_TABLE 3. Data was processed using Thermo XCalibur 2.2.44 Quanbrowser extracting the fragments from the transitions as given in SI_TABLE 3. The results are shown in SI_TABLE 4.

Quantitative LC-MS/MS analysis of m^5C and m^6A

The RNA digests (0.5 µg in 50 µl) were digested as described with the exception of the addition of 12.5 µl of a 100 nM solution of [$^{15}N_2{}^{13}C$]-rC, [$^{15}N_5$]-rA, [5,5,5,6-D_4]-m^5C and [6,6,6-D_3]-m^6A (25 nM final concentration). A dilution series of rC, rA, m^5C and m^6A was then prepared in the range of 0.75 – 11,250 nM containing 25 nM of the SIL internal standards [$^{15}N_2{}^{13}C$]-rC, [$^{15}N_5$]-rA, [5,5,5,6-D_4]-m^5C and [6,6,6-D_3]-m^6A. Calibration standards and samples were measured at 8 µl injections using a Thermo Ultimate 3000 UHPLC system equipped with a Waters HSS-T3 column (2.1 x 100 mm, 1.8 µm particle size) and coupled to a Thermo Qexactive hybrid mass spectrometer. LC conditions were as follows: H_2O – MeCN solvent system containing 0.1 % formic acid; 0 – 100 % multistep gradient over 5 minutes (0 – 30 % 3 min; 100 % 4 min; 100 % 5 min) and 1 minute column regeneration time. LC-MS/HRMS was performed using a HESI source (4 kV, capillary T: 350 ^oC, sheath gas: 30, auxiliary gas: 2, heater T: 350 ^oC. The machine was operated in MRM mode; resolution: 35,000, isolation window: 3.0 m/z, NCE: 10 and the following inclusions: rC m/z: 244; [$^{15}N_2{}^{13}C$]-rC m/z: 247; rA m/z: 268; [$^{15}N_5$]-rA m/z: 273; m^5C m/z: 258; [5,5,5,6-D_4]-m^5C m/z: 262; m^6A m/z: 282; [6,6,6-D_3]-m^6A m/z: 285. Data was processed using Thermo XCalibur 2.2.44 Quanbrowser.

***C. elegans* husbandry**

C. elegans were grown under standard conditions at 20 °C unless otherwise indicated. The wild-type strain was var. Bristol N2.[2]

Culturing *E. coli* on ^{12}C or ^{13}C D-glucose

Starter cultures of E. coli HT115 strain were grown over-night in M9 minimal media (100ml of 10X M9 salts (5.8% w/v Na_2HPO_4, 3% w/v KH_2PO_4, 0.5% w/v NaCl, 1% w/v NH_4Cl), 10 ml 20 % glucose (^{12}C or ^{13}C), 1 mM $MgSO_4$ up to 1l ddH_2O). Starter cultures were used for growing fresh *E. coli* HT115 cultures in M9 minimal media to an O.D - 0.8-1.0.

^{13}C labeling of *C. elegans* using ^{13}C labeled *E. coli* as food source

10 ml/plate E. coli HT115 cultures were grown to O.D - 0.8-1.0, pelleted and re-suspended in 500 µl M9 minimal media. Re-suspended bacteria were seeded to NGM-N agarose plates (1.2 % agarose, 0.3 % NaCl, 1mM $CaCl_2$, 1mM $MgSO_4$, 1 mM KPO4, 5 µg/ml cholesterol) and the plates were left to dry over-night. 10 larval stage 1 animals were placed on seeded NGM-N agarose plates with the labeled bacteria and left to grow for 2 generations. Adult F1 generation animals were bleached to obtain a synchronous population of F2 generation animals. F2 animals were placed on freshly seeded NGM-N plates with labeled bacteria and grown to adult stage. Adult animals were washed off the plates and pelleted before DNA and RNA isolation.

Heat stress and starvation of *C. elegans* larvae

Synchronized population of L1 animals were grown until young adult stage. For heat stress, animals were transferred to 37 ^{o}C incubators for 4 hrs with food. After 4hrs, animals were washed off the plates, cleaned from bacteria by washing in M9 buffer 3X, and stored in TRIsure *(Bioline*) reagent for subsequent RNA isolation. For starvation experiments, young adult stage animals were washed off the plates using M9 buffer and plated on either food plates for control or on empty plates for starvation. Animals were left at 20 ^{o}C for 4 hrs. After 4 hrs, animals were washed off the plates, cleaned by washing in M9 buffer 3X and stored in TRIsure reagent for RNA isolation. For rescue experiments, control and starved animals were plated on plates with food and left for 8 hrs at 20 ^{o}C. Due to 8hrs difference in ageing, it is essential to have age matched control animals along side the rescued animals to avoid changes in that might occur due to ageing process rather than starvation and rescue.

C. elegans disruption for total RNA isolation

Animals were disrupted by bead beating in TRIsure (*Bioline*) on a Precellys 24 (*Bertin Technologies*) instrument using 0.7 mm zirconia beads (*BioSpec*) and 3 x 20 second pulses at 6500 rpm with 20 seconds break in between. The lysate was removed from the beads and total RNA was extracted according to the manufacturer's instructions (see total RNA isolation).

Total RNA isolation

0.2 ml of chloroform was added per ml of TRIsure used. The sample was shaken vigorously for 15 seconds, allowed to stand for 5 minutes at room temperature and centrifuged at 12,000 x g for 15 minutes at 4 °C. The aqueous phase was transferred to a fresh tube and 0.5 ml of 2-propanol was added per ml of TRIsure used in the sample preparation. The sample was allowed to stand 10 minutes at room temperature and centrifuged at 12,000 x g for 10 minutes at 4 °C to precipitate the RNA at the bottom and side of the tube. The supernatant was removed and the RNA pellet was washed with 75 % ethanol, air-dried and redissovled in nuclease-free water. Total RNA was then purified using RNA Clean & Concentrator (*Zymo Research*).

Small/large RNA fractionation

Total RNA was fractionated into small (< 200 nt) and large (> 200 nt) RNAs either using the Quick-RNA MiniPrep kit (*Zymo Research*) according to the manufacturer's instructions or gel electrophoresis. In the ladder, total RNA and a low range ssRNA ladder (*NEB*) were run on a 15 % Novex TBE-urea gel (*Life Technologies*) for 60 min at 180 V. The ladder was excised from the gel, stained with CYBRGold and visualised under UV. Gel sections of the corresponding RNA bands of interest were excised from an unstained gel and RNA was eluted by overnight agitation at 4 °C in gel elution buffer (0.3 M NaCl, 0.25 % SDS, 1 mM EDTA (pH 8)). RNA was precipitated using isopropanol, washed with 75 % EtOH, air-dried and redissolved in water.

RNA digestion for LC-MS/MS analysis

Digestion enzyme master mix was prepared by combining benzonase (250 U/µl, 0.625 µl, *Sigma Aldrich*), phosphodiesterase I from Crotalus adamanteus venom (10 mU/µl, 10 µl, *Sigma Aldrich*) and Antarctic phosphatase (5 U/µl, 20 µl, *NEB*). Aqueous solutions of total RNA (1 µg in 13.25 µl final volume) were mixed with 5x digestion buffer (5 µl, Tris-HCl pH 8 (20 mM), $MgCl_2$ (20 mM), NaCl (100 mM)) and digestion enzyme stock solution (0.5 µl) and water (6.25 µL), followed by incubation at 37 °C for 14 h. The nucleoside mixture was subsequently cleaned-up by filtration over Amicon Ultra 0.5 ml (10 kDa MWCO, Merck-Milipore) spin columns.

Analysis of codon usage

Coding sequences for all genes of *C. elegans* were downloaded from Ensembl Biomart release 84 (WBcel235).[3] For each gene, the longest valid coding sequence was taken to represent a gene's codon usage. Differential expression analysis was performed between the control and the starvation treatment, and the control and the heatshock treatment using DESeq2 using three biological replicates.[4] Genes were called differentially expressed if their adjusted p-value (Wald test) was below 0.01. Next, codon usage was summed across differentially expressed, upregulated genes separately in both treatment and control, with each gene's codon usage weighted by its gene expression (variance stabilized count). We now wanted to assess whether codons of tRNAs with mcm^5s^2U modifications were significantly enriched or depleted under heatshock treatment. This was done by calculating the difference in codon usage between control and treatment. We then computed the prediction interval based on the codon usage difference of the remaining 58 codons using the Vysochanskij–Petunin inequality with $\lambda=3$.[4] RNA-Seq data are available in the ArrayExpress database (www.ebi.ac.uk/arrayexpress) under accession number E-MTAB-4940.

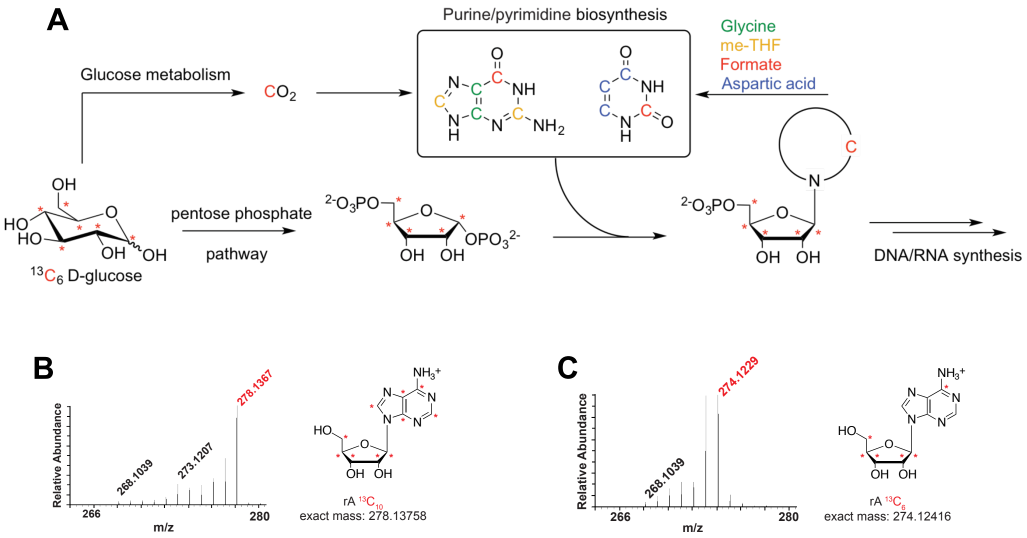

Figure S1: ^{13}C-labeling of the *C. elegans* transcriptome.
(A) Glucose metabolism influences the biosynthesis of nucleobases by producing CO_2, which site-specifically introduces ^{13}C labels into RNA nucleosides (red colored C) to create a $^{13}C_6$ fingerprint. Introduction of ^{13}C into other positions is suppressed through addition of unlabeled amino acids. This inhibits their *de novo* synthesis from $^{13}C_6$-glucose. (B-C) Representative HRMS spectra for adenosine (rA) obtained from ^{13}C-labelled nematodes that have either been grown in the absence (B) or presence (C) of amino acids.

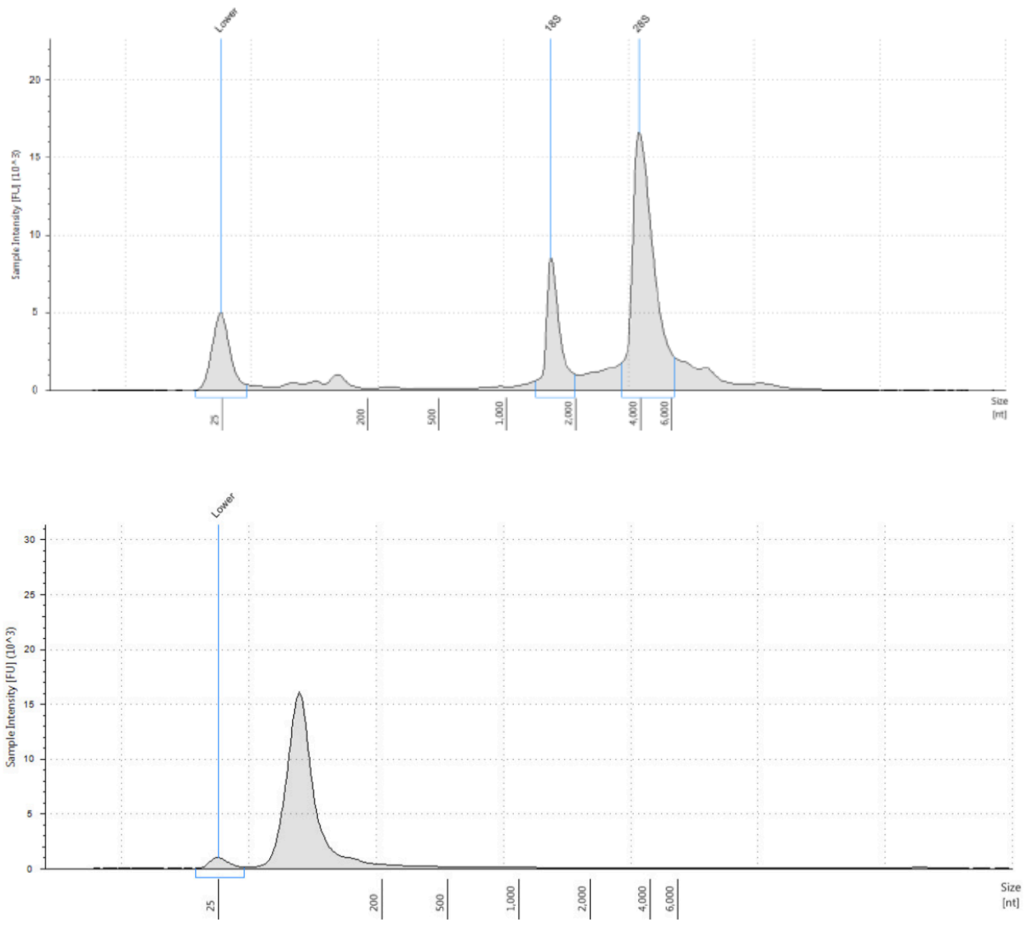

Figure S2: Representative Agilent TapeStation electropherograms of large (> 200 nt) and small (< 200 nt) fractions.
The large (top) and small (bottom) fractions were obtained from *C. elegans* total RNA fractionation using the Quick-RNA MiniPrep kit (*Zymo Research*).

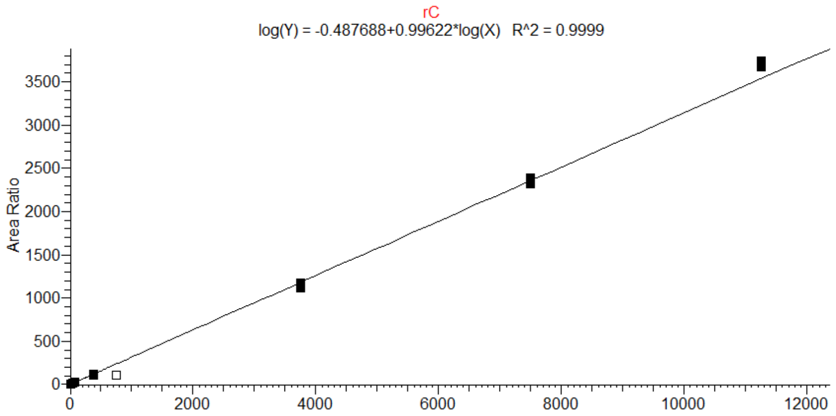

Figure S3: Calibration curve used for the quantification of rC in *C. elegans* small and large RNA samples.
Calibration standards were spiked with a known and fixed amount of $^{13}C,^{15}N_2$-cytidine. As a result, the detector responses (peak surface areas) for both unlabeled and SIL ribonucleosides could be extracted and plotted as the ration against the concentration (in nM) of the unlabeled nucleoside standard to obtain the above calibration curve.

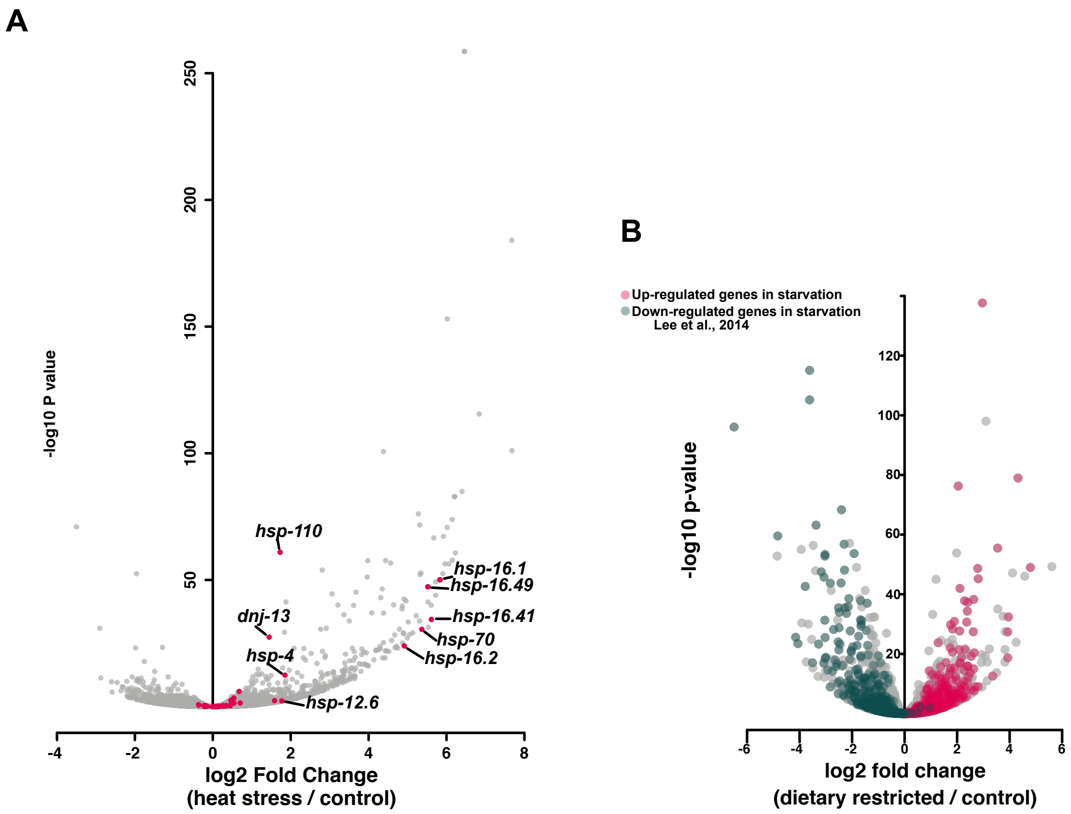

Figure S4: RNA-Seq data analysis of heat-shocked and starved animals.
Log2 fold change of genes (treated/wild type, x-axis) plotted against –log 10 p-value for each gene (3 biological replicates). (A) Heat-shocked induced known heat shock factors are highlighted by red dots and labeled. (B) Genes differentially expressed in starved animals are plotted in grey (this study) and up and down regulated starvation genes are marked in green and red.[5]

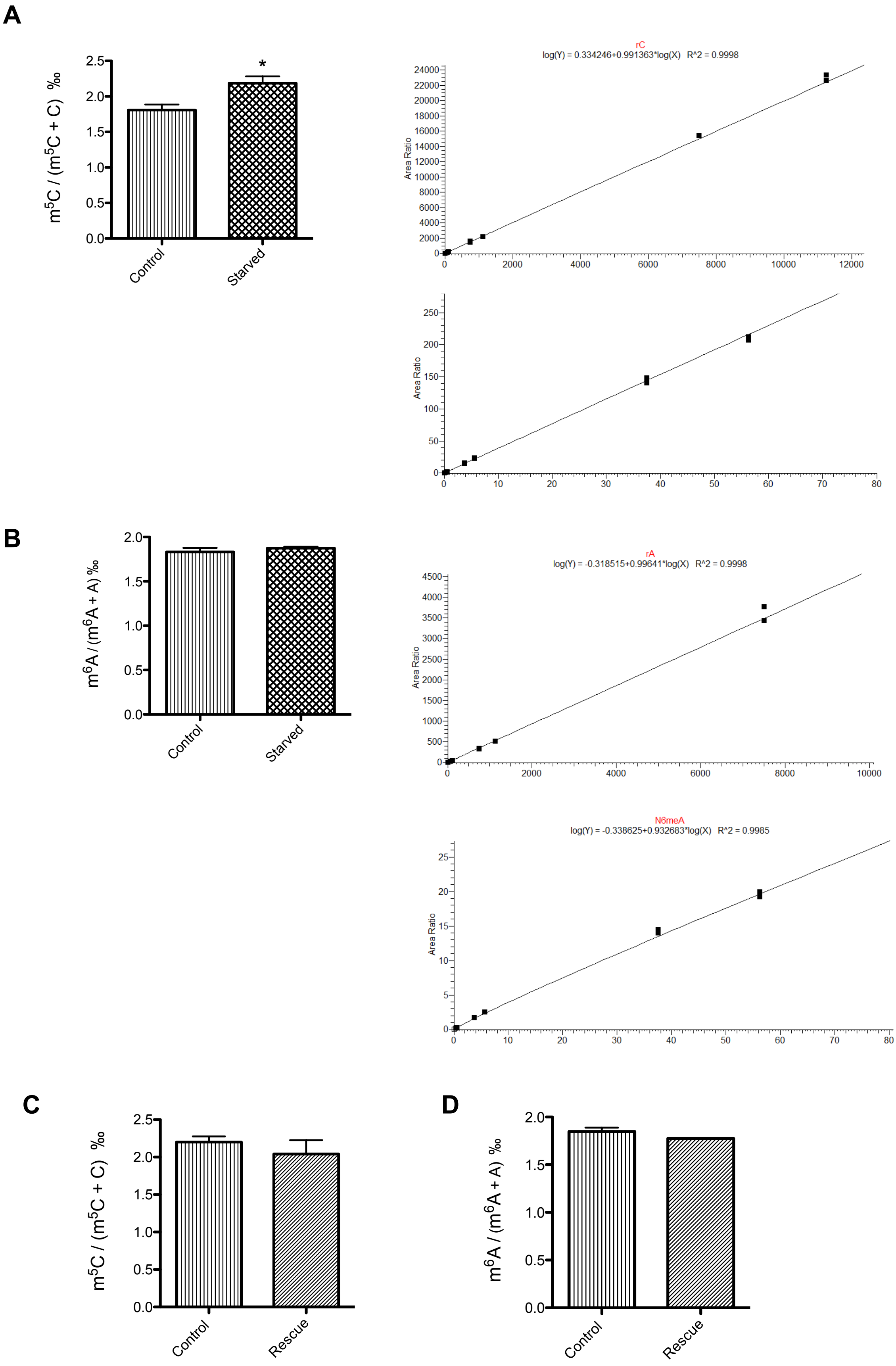

Figure S5: Absolute quantification of RNA modifications.
Absolute quantitation of m^{5}C (A) and m^{6}A (B) on the large RNA fraction of control and starved *C. elegans* and corresponding calibration curves. Absolute quantitation of m^{5}C (C) and m^{6}A (D) on the large fraction of control and dietary rescued *C. elegans*.

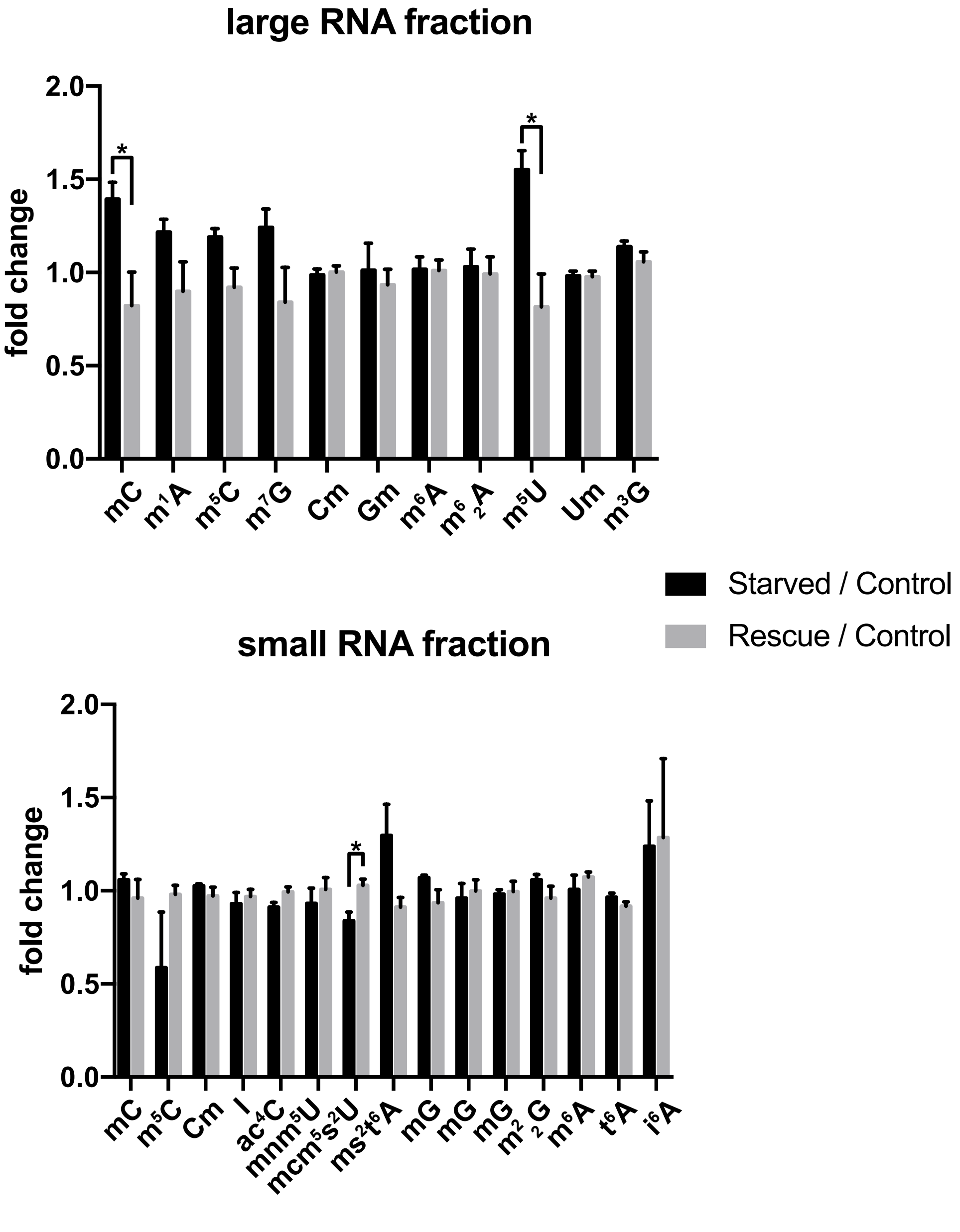

Figure S6: Dynamic changes of RNA modifications in *C. elegans*.
Fold change of RNA modifications (starved/control in black, rescue/control in grey) are shown for large RNA fraction (upper panel) and small RNA fraction (lower panel) (error bars indicate SEM, * p-value ≤ 0.05).

References

(1) Huber, S. M.; van Delft, P.; Mendil, L.; Bachman, M.; Smollett, K.; Werner, F.; Miska, E. A.; Balasubramanian, S. *ChemBioChem* **2015**, *16* (5), 752.

(2) Brenner, S. *Genetics* **1974**, *77* (1), 71.

(3) Flicek, P.; Amode, M. R.; Barrell, D.; Beal, K.; Billis, K.; Brent, S.; Carvalho-Silva, D.; Clapham, P.; Coates, G.; Fitzgerald, S.; Gil, L.; Girón, C. G.; Gordon, L.; Hourlier, T.; Hunt, S.; Johnson, N.; Juettemann, T.; Kähäri, A. K.; Keenan, S.; Kulesha, E.; Martin, F. J.; Maurel, T.; McLaren, W. M.; Murphy, D. N.; Nag, R.; Overduin, B.; Pignatelli, M.; Pritchard, B.; Pritchard, E.; Riat, H. S.; Ruffier, M.; Sheppard, D.; Taylor, K.; Thormann, A.; Trevanion, S. J.; Vullo, A.; Wilder, S. P.; Wilson, M.; Zadissa, A.; Aken, B. L.; Birney, E.; Cunningham, F.; Harrow, J.; Herrero, J.; Hubbard, T. J. P.; Kinsella, R.; Muffato, M.; Parker, A.; Spudich, G.; Yates, A.; Zerbino, D. R.; Searle, S. M. J. *Nucleic Acids Res.* **2014**, *42* (D1), 749.

(4) Love, M. I.; Huber, W.; Anders, S. *Genome Biol.* **2014**, *15* (12), 550.

(5) Lee, D.; Hwang, W.; Artan, M.; Jeong, D. E.; Lee, S. J. *Aging Cell* **2015**, *14* (1), 8.

m/z observed unlabeled	m/z observed labeled	Δ m/z observed (m/z labeled - m/z unlabeled)	retention time [min]	m/z theoretical unlabeled	m/z theoretical labeled	mass acurracy unlabeled [ppm]	mass accuracy labeled [ppm]	assigned residue
410.1764081	416.1965081	6.0201	1.48					
433.1296918	439.1497918	6.0201	1.48					C [M+tris]
465.0777166	471.0978166	6.0201	1.48					
471.1829712	477.2030712	6.0201	1.48					
476.1995265	482.2196265	6.0201	1.48					
358.1454818	363.1622318	5.01675	1.50					
328.1133622	333.1301122	5.01675	1.50					
258.1082604	264.1283604	6.0201	1.62	258.10897	264.1291	-2.7	-2.8	methyl cytosine
282.1194154	288.1395154	6.0201	2.02	282.120203	288.140333	-2.8	-2.8	methyl adenosine
258.1082604	264.1283604	6.0201	2.13	258.10897	264.1291	-2.7	-2.8	methyl cytosine
272.1349867	278.1550867	6.0201	2.23					
479.0173624	485.0374624	6.0201	2.39					
488.0121717	493.0289217	5.01675	2.39					
427.0195138	433.0396138	6.0201	2.39					
489.0187778	495.0388778	6.0201	2.40					
426.1499456	431.1666956	5.01675	2.40					
298.1142207	304.1343207	6.0201	3.13	298.115118	304.135248	-3.0	-3.0	methyl guanosine
379.1815706	384.1983206	5.01675	3.49					
258.1082604	264.1283604	6.0201	3.49	258.10897	264.1291	-2.7	-2.8	methyl cytosine
288.1184299	294.1385299	6.0201	3.60					
259.0922735	265.1123735	6.0201	3.68	259.092986	265.113116	-2.7	-2.8	methyl uridine or pseudouridine
290.0854288	296.1055288	6.0201	3.83					
268.103738	274.123838	6.0201	3.89	268.104553	274.124683	-3.0	-3.1	adenosine
284.0985921	290.1186921	6.0201	4.02	284.099468	290.119598	-3.1	-3.1	guanosine
252.1087611	258.1288611	6.0201	4.10					
300.0933937	306.1134937	6.0201	4.27					
314.1081642	320.1282642	6.0201	4.38					
404.0507062	410.0708062	6.0201	4.44					
487.1031344	492.1198844	5.01675	4.44					
259.0922735	265.1123735	6.0201	4.44	259.092986	265.113116	-2.7	-2.8	methyl uridine or pseudouridine
395.0558636	401.0759636	6.0201	4.44					
284.0985921	290.1186921	6.0201	4.52	284.099468	290.119598	-3.1	-3.1	guanosine
288.1119214	294.1320214	6.0201	4.52					
289.1150854	294.1318354	5.01675	4.52					
312.1297341	318.1498341	6.0201	4.65	312.130768	318.150898	-3.3	-3.3	dimethyl guanosine
281.0741226	287.0942226	6.0201	4.76	281.074956	287.09454	-3.0	-1.1	methyl uridine or pseudouridine
395.0559089	400.0726589	5.01675	4.76					
404.0507062	410.0708062	6.0201	4.76					
359.0163837	365.0364837	6.0201	4.77					
375.0972998	381.1173998	6.0201	4.76					
396.0581735	402.0782735	6.0201	4.76					
410.987492	417.007592	6.0201	4.75					
487.1031257	493.1232257	6.0201	4.76					
493.0328492	499.0529492	6.0201	4.77					
320.0957776	326.1158776	6.0201	4.80					
298.1142207	304.1343207	6.0201	4.80	298.115118	304.135248	-3.0	-3.0	methyl guanosine
419.1877516	425.2078516	6.0201	4.80					
282.1194154	288.1395154	6.0201	4.85	282.120203	288.140333	-2.8	-2.8	methyl adenosine
320.0957776	326.1158776	6.0201	4.89					
419.1877516	425.2078516	6.0201	4.89					
298.1142207	304.1343207	6.0201	4.89	298.115118	304.135248	-3.0	-3.0	methyl guanosine
309.1385686	314.1553186	5.01675	4.93					
326.1453496	332.1654496	6.0201	4.95	326.146418	332.166548	-3.3	-3.3	trimethyl guanosine
286.1032783	292.1233783	6.0201	4.98	286.103885	292.124015	-2.1	-2.2	acetyl cytosine
312.1297341	318.1498341	6.0201	5.06	312.130768	318.150898	-3.3	-3.3	dimethyl guanosine
327.1659783	332.1827283	5.01675	5.08					
364.6713758	370.6914758	6.0201	5.13					
437.2067422	442.2234922	5.01675	5.16					
413.140965	418.157715	5.01675	5.17	413.142061	418.15831	-2.7	-1.4	threonylcarbamoyl adenosine
296.1350885	302.1551885	6.0201	5.17	296.135853	302.155983	-2.6	-2.6	dimethyl adenosine
333.0743072	338.0910572	5.01675	5.20	333.075621	338.09187	-3.9	-2.4	5-methoxycarbonylmethyl-2-thiouridine
284.0985921	290.1186921	6.0201	5.21	284.099468	290.119598	-3.1	-3.1	guanosine
405.1722651	411.1923651	6.0201	5.24					
284.0985921	290.1186921	6.0201	5.24	284.099468	290.119598	-3.1	-3.1	guanosine
323.1284227	329.1485227	6.0201	5.29					
284.0985921	290.1186921	6.0201	5.42	284.099468	290.119598	-3.1	-3.1	guanosine
493.1976315	499.2177315	6.0201	5.42					
336.1656915	342.1857915	6.0201	5.64	336.167153	342.187283	-4.3	-4.4	isopentyl adenosine
282.1194154	288.1395154	6.0201	6.01	282.120203	288.140333	-2.8	-2.8	methyl adenosine

Quantification of cytidine (rC) in RNA samples

Equation obtained from standards 1-10:
log(Y) = -0.487688+0.99622*log(X)   R^2 = 0.9999

sample name	rC area	internal standard area	area ratio (rC area/ internal standard area)	rC concentration (nM)	calculated rC concenration using above equation	retention time
Standard 1	237812	1382157	0.172	0.375	0.528	1.24
Standard 2	329387	1381138	0.238	0.750	0.732	1.26
Standard 3	1839477	1347243	1.365	3.750	4.220	1.26
Standard 4	3364442	1368291	2.459	7.500	7.617	1.26
Standard 5	15049124	1230704	12.228	37.500	38.109	1.29
Standard 6	26932833	1142205	23.580	75.000	73.669	1.29
Standard 7	106484660	911803	116.785	375.000	367.087	1.28
Standard 8	157846109	1449157	108.923	750.000	342.284	1.28
Standard 9	748467099	649937	1151.599	3750.000	3651.372	1.28
Standard 10	1264432703	543596	2326.050	7500.000	7394.902	1.26
Standard 11	1806588238	489484	3690.801	11250.000	11754.244	1.24
Heat 1 Large RNA	161463179	838598	192.539	unknown	606.355	1.28
Heat 2 Large RNA	294551056	739095	398.529	unknown	1258.538	1.26
Heat 3 Large RNA	187421353	835325	224.369	unknown	707.006	1.28
Fed Fed (Control) 1 Large RNA	208215411	851567	244.509	unknown	770.718	1.26
Fed Fed (Control) 2 Large RNA	200840542	851849	235.770	unknown	743.070	1.28
Fed Fed (Control) 3 Large RNA	182324249	834202	218.561	unknown	688.635	1.26
Fed Starve 1 Large RNA	240921741	773034	311.657	unknown	983.282	1.26
Fed Starve 2 Large RNA	224313409	773929	289.837	unknown	914.187	1.26
Fed Starve 3 Large RNA	235896683	761716	309.691	unknown	977.056	1.26
Fed Fed Fed (Control) 1 Large RNA	344115388	672610	511.612	unknown	1617.181	1.26
Fed Fed Fed (Control) 2 Large RNA	386498804	684278	564.827	unknown	1786.061	1.26
Fed Fed Fed (Control) 3 Large RNA	400099194	683202	585.624	unknown	1852.076	1.24
Fed Starve Fed (Rescue) 1 Large RNA	274225410	736292	372.441	unknown	1175.851	1.24
Fed Starve Fed (Rescue) 2 Large RNA	491732515	680073	723.059	unknown	2288.555	1.24
Fed Starve Fed (Rescue) 3 Large RNA	193378198	814937	237.292	unknown	747.885	1.26
Heat 1 Small RNA	138084867	860213	160.524	unknown	505.182	1.28
Heat 2 Small RNA	131456766	863602	152.219	unknown	478.949	1.26
Heat 3 Small RNA	133052457	888481	149.753	unknown	471.159	1.26
Fed Fed (Control) 1 Small RNA	130613141	912373	143.158	unknown	450.333	1.26
Fed Fed (Control) 2 Small RNA	132344083	885278	149.494	unknown	470.343	1.24
Fed Fed (Control) 3 Small RNA	143775915	882315	162.953	unknown	512.855	1.26
Fed Starve 1 Small RNA	98021427	925248	105.941	unknown	332.878	1.26
Fed Starve 2 Small RNA	158984598	866233	183.536	unknown	577.895	1.24
Fed Starve 3 Small RNA	119367188	914842	130.478	unknown	410.303	1.26
Fed Fed Fed (Control) 1 Small RNA	128076923	896991	142.785	unknown	449.156	1.26
Fed Fed Fed (Control) 2 Small RNA	153626657	851604	180.397	unknown	567.974	1.28
Fed Fed Fed (Control) 3 Small RNA	152618283	855569	178.382	unknown	561.608	1.28
Fed Starve Fed (Rescue) 1 Small RNA	98763720	918181	107.565	unknown	338.000	1.28
Fed Starve Fed (Rescue) 2 Small RNA	160457107	846142	189.634	unknown	597.170	1.28
Fed Starve Fed (Rescue) 3 Small RNA	135920583	877810	154.841	unknown	487.229	1.28

SI_TABLE2

Analyte	Inclusion [M+H]+	Transition MS/HRMS (±5ppm)
		exact mass [M+H]+
methyl cytidine	258	126.06619
methyl cytidine 13C6	264	127.06954
Cm	258	112.05054
Cm 13C6	264	113.05389
ac4C	286	154.0611
ac4C 13C6	292	155.06446
I	269	137.04579
I 13C6	275	138.04914
meI	283	151.06144
meI 13C6	289	152.06479
ms2t6 A	459	327.087
ms2t6 A 13C6	465	328.09035
mnm5 Um	288	156.07675
mnm5 Um 13C6	294	157.08011
mcm5s2 U	333	201.03284
mcm5s2 U 13C6	339	202.0319
mchm5 U	333	201.0506
mchm5 U 13C6	339	202.05395
methyl guanosine	298	166.07234
methyl guanosine 13C6	304	167.07569
Gm	298	152.05669
Gm 13C6	304	153.06004
dimethyl guanosine	312	180.08799
dimethyl guanosine 13C6	318	181.09134
trimethyl guanosine	326	194.10364
trimethyl guanosine 13C6	332	195.10699
methyl adenosine	282	150.07742
methyl adenosine 13C6	288	151.08078
Am	282	136.06177
Am 13C6	288	137.06513
dimethyl adenosine	296	164.09307
dimethyl adenosine 13C6	302	165.09643
t6A	413	281.09928
t6A 13C6	419	282.10263
i6A	336	204.12437
i6A 13C6	342	205.12773
methyl uridine	258	127.0502
methyl uridine 13C6	264	128.05356
Um	258	113.03455
Um 13C6	264	114.03791

small + large

small RNA

SI TABLE3

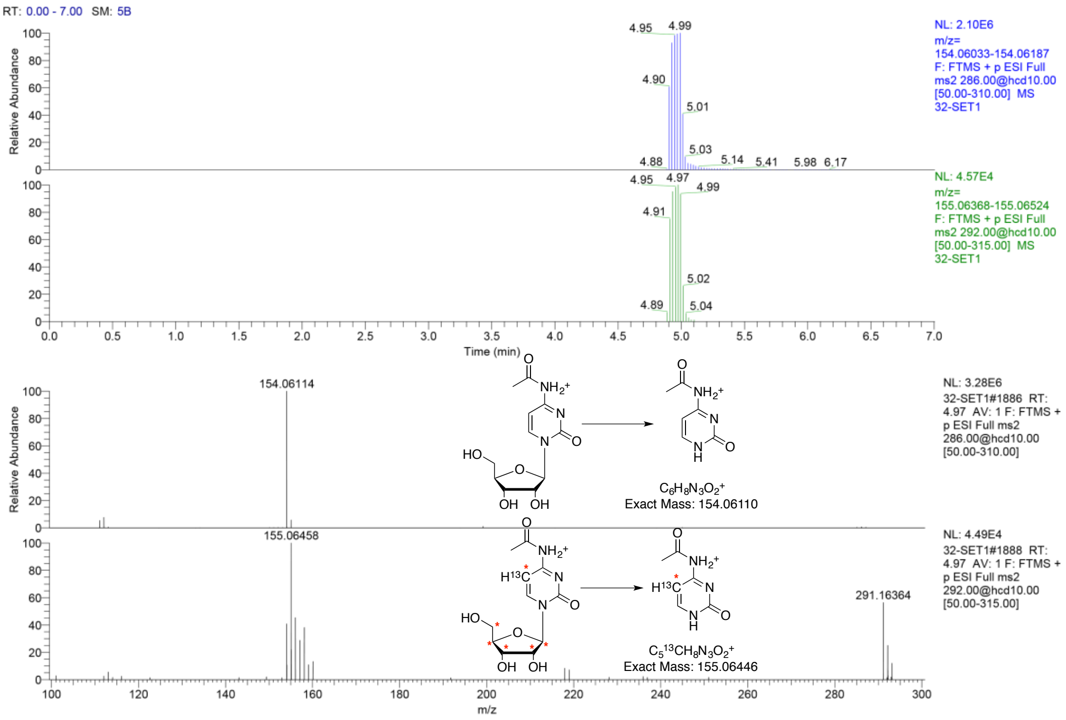
RT: 0.00 - 7.00 SM: 5B
NL: 2.10E6
m/z= 154.06033-154.06187
F: FTMS + p ESI Full ms2 286.00@hcd10.00 [50.00-310.00] MS 32-SET1
NL: 4.57E4
m/z= 155.06368-155.06524
F: FTMS + p ESI Full ms2 292.00@hcd10.00 [50.00-315.00] MS 32-SET1
Relative Abundance
Time (min)
154.06114
155.06458
291.16364
C6H8N3O2+
Exact Mass: 154.06110
C5 13CH8N3O2+
Exact Mass: 155.06446
NL: 3.28E6
32-SET1#1886 RT: 4.97 AV: 1 F: FTMS + p ESI Full ms2 286.00@hcd10.00 [50.00-310.00]
NL: 4.49E4
32-SET1#1888 RT: 4.97 AV: 1 F: FTMS + p ESI Full ms2 292.00@hcd10.00 [50.00-315.00]
m/z

Filename	Sample Type	Area	ISTD Area	Area Ratio	Average Ratio	Fold Change	p-value

SMALL RNA FRACTION

Component Name
mC (methylated cytidine)

Filename	Sample Type	Area	ISTD Area	Area Ratio	Average Ratio	Fold Change	p-value
32-SET1	Heat 1	23616478	923428	25.575			
33-SET1	Heat 2	29709040	1001068	29.677			
34-SET1	Heat 3	28076315	1041457	26.959	27.404	0.00	0.9770
35-SET1	Fed Fed (Control) 1	28252205	1043759	27.068			
36-SET1	Fed Fed (Control) 2	30158131	1046616	28.815			
37-SET1	Fed Fed (Control) 3	27460158	1037858	26.459	27.447		
38-SET1	Fed Starve 1	28393328	936264	30.326			
39-SET1	Fed Starve 2	27776478	928707	29.909			
40-SET1	Fed Starve 3	23718486	878253	27.006	29.080	0.06	0.2736
41-SET1	Fed Fed Fed (Control) 1	33493482	1089868	30.732			
42-SET1	Fed Fed Fed (Control) 2	30214394	998489	30.260			
43-SET1	Fed Fed Fed (Control) 3	30476369	943908	32.287	31.093		
44-SET1	Fed Starve Fed (Rescue) 1	28319218	957551	29.575			
45-SET1	Fed Starve Fed (Rescue) 2	30952011	902982	34.278			
46-SET1	Fed Starve Fed (Rescue) 3	26030975	1021166	25.491	29.781	-0.04	0.6606

63154 **SD**
983425 **Average**
7% **Relative SD**

Component Name
m5C (5-methylcytidine)

Filename	Sample Type	Area	ISTD Area	Area Ratio	Average Ratio	Fold Change	p-value
32-SET1	Heat 1	130231294	4460914	29.194			
33-SET1	Heat 2	139192620	3983869	34.939			
34-SET1	Heat 3	137573664	4321382	31.836	31.989	0.12	0.1726
35-SET1	Fed Fed (Control) 1	118614519	4129846	28.721			
36-SET1	Fed Fed (Control) 2	120377635	4228911	28.465			
37-SET1	Fed Fed (Control) 3	121016987	4258418	28.418	28.535		
38-SET1	Fed Starve 1	106018836	4698353	22.565			
39-SET1	Fed Starve 2	117454665	4229177	27.772			
40-SET1	Fed Starve 3	56843454	NF	NF	25.169	-0.12	0.4189
41-SET1	Fed Fed Fed (Control) 1	123377630	4377098	28.187			
42-SET1	Fed Fed Fed (Control) 2	124421313	4453449	27.938			
43-SET1	Fed Fed Fed (Control) 3	119334656	4270125	27.946	28.024		
44-SET1	Fed Starve Fed (Rescue) 1	101464410	3365306	30.150			
45-SET1	Fed Starve Fed (Rescue) 2	118780216	4713204	25.202			
46-SET1	Fed Starve Fed (Rescue) 3	122541184	4508741	27.179	27.510	-0.02	0.7552

332956 **SD**
4285628 **Average**
8% **Relative SD**

Component Name
Cm (2'-O-methylcytidine)

Filename	Sample Type	Area	ISTD Area	Area Ratio	Average Ratio	Fold Change	p-value
32-SET1	Heat 1	50525949	1779752	28.389			
33-SET1	Heat 2	52969728	1663352	31.845			
34-SET1	Heat 3	48956735	1750076	27.974	29.403	0.01	0.8240
35-SET1	Fed Fed (Control) 1	50913645	1748786	29.114			
36-SET1	Fed Fed (Control) 2	51983619	1765886	29.438			
37-SET1	Fed Fed (Control) 3	49789836	1733665	28.719	29.090		
38-SET1	Fed Starve 1	55749005	1830449	30.456			
39-SET1	Fed Starve 2	50327951	1693807	29.713			
40-SET1	Fed Starve 3	52435746	1779865	29.461	29.877	0.03	0.1052
41-SET1	Fed Fed Fed (Control) 1	50873888	1694327	30.026			
42-SET1	Fed Fed Fed (Control) 2	50528594	1738187	29.070			
43-SET1	Fed Fed Fed (Control) 3	51476976	1722083	29.892	29.663		
44-SET1	Fed Starve Fed (Rescue) 1	57085274	1859940	30.692			
45-SET1	Fed Starve Fed (Rescue) 2	52342750	1772835	29.525			
46-SET1	Fed Starve Fed (Rescue) 3	45798067	1745115	26.244	28.820	-0.03	0.5948

50732 **SD**
1751875 **Average**
3% **Relative SD**

Component Name
I (inosine)

Filename	Sample Type	Area	ISTD Area	Area Ratio	Average Ratio	Fold Change	p-value
32-SET1	Heat 1	6156690	99429	61.921			
33-SET1	Heat 2	6312782	89293	70.697			
34-SET1	Heat 3	5622369	84763	66.331	66.316	0.02	0.7796
35-SET1	Fed Fed (Control) 1	6008368	96240	62.431			
36-SET1	Fed Fed (Control) 2	5849941	79554	73.534			
37-SET1	Fed Fed (Control) 3	5624453	96640	58.200	64.722		
38-SET1	Fed Starve 1	6829196	116547	58.596			
39-SET1	Fed Starve 2	5848152	96530	60.584			
40-SET1	Fed Starve 3	6509412	108452	60.021	59.734	-0.08	0.3893
41-SET1	Fed Fed Fed (Control) 1	6152955	97699	62.979			
42-SET1	Fed Fed Fed (Control) 2	5925375	92689	63.927			
43-SET1	Fed Fed Fed (Control) 3	6306879	101902	61.892	62.933		
44-SET1	Fed Starve Fed (Rescue) 1	6993813	106317	65.783			
45-SET1	Fed Starve Fed (Rescue) 2	6086129	100837	60.356			
46-SET1	Fed Starve Fed (Rescue) 3	5860885	102880	56.968	61.036	-0.03	0.5399

9251 **SD**
97985 **Average**
10% **Relative SD**

Component Name
ac4C (N4-acetylcytidine)

Filename	Sample Type	Area	ISTD Area	Area Ratio	Average Ratio	Fold Change	p-value
32-SET1	Heat 1	14199768	297633	47.709			
33-SET1	Heat 2	13648181	273668	49.871			
34-SET1	Heat 3	13484079	290763	46.375	47.985	0.03	0.3737
35-SET1	Fed Fed (Control) 1	13628818	285600	47.720			
36-SET1	Fed Fed (Control) 2	14258899	309210	46.114			
37-SET1	Fed Fed (Control) 3	12355576	265329	46.567	46.800		
38-SET1	Fed Starve 1	13343082	314550	42.420			
39-SET1	Fed Starve 2	12242794	275708	44.405			
40-SET1	Fed Starve 3	11348642	274854	41.290	42.705	-0.09	0.0279
41-SET1	Fed Fed Fed (Control) 1	11966900	251922	47.502			
42-SET1	Fed Fed Fed (Control) 2	14107519	307861	45.824			
43-SET1	Fed Fed Fed (Control) 3	12532180	300775	41.666	44.998		
44-SET1	Fed Starve Fed (Rescue) 1	14373700	305066	47.117			
45-SET1	Fed Starve Fed (Rescue) 2	13075310	301119	43.422			
46-SET1	Fed Starve Fed (Rescue) 3	11721671	270006	43.413	44.651	-0.01	0.8792

18803 **SD**
288271 **Average**
7% **Relative SD**

Filename	Sample Type	Area	ISTD Area	Area Ratio	Average Ratio	Fold Change	p-value

SMALL RNA FRACTION

Component Name

mchm5U (5-(carboxyhydroxymethyl)uridine methyl ester)

Filename	Sample Type	Area	ISTD Area	Area Ratio	Average Ratio	Fold Change	p-value
32-SET2	Heat 1	422231	4881	86.497			
33-SET2	Heat 2	500194	6811	73.444			
34-SET2	Heat 3	467074	7883	59.250	73.064	-0.04	0.8108
35-SET2	Fed Fed (Control) 1	452753	6167	73.411			
36-SET2	Fed Fed (Control) 2	497620	5520	90.152			
37-SET2	Fed Fed (Control) 3	468388	7314	64.040	75.868		
38-SET2	Fed Starve 1	408303	3498	87.769			
39-SET2	Fed Starve 2	405776	5653	71.785			
40-SET2	Fed Starve 3	399521	5199	71.159	76.904	0.01	0.9178
41-SET2	Fed Fed Fed (Control) 1	435881	7618	57.216			
42-SET2	Fed Fed Fed (Control) 2	444007	3447	65.000			
43-SET2	Fed Fed Fed (Control) 3	419099	5348	78.370	66.862		
44-SET2	Fed Starve Fed (Rescue) 1	445586	4580	74.266			
45-SET2	Fed Starve Fed (Rescue) 2	427984	5401	79.247			
46-SET2	Fed Starve Fed (Rescue) 3	404510	8553	47.294	66.936	0.00	0.9953

1526 **SD**
5858 **Average**
26% **Relative SD**

Component Name

mnm5U (5-methylaminomethyluridine)

Filename	Sample Type	Area	ISTD Area	Area Ratio	Average Ratio	Fold Change	p-value
32-SET2	Heat 1	2047135	69182	29.591			
33-SET2	Heat 2	2526467	62282	40.565			
34-SET2	Heat 3	2192720	70821	30.962	33.706	-0.01	0.9363
35-SET2	Fed Fed (Control) 1	2555318	82927	30.814			
36-SET2	Fed Fed (Control) 2	2692860	73107	36.835			
37-SET2	Fed Fed (Control) 3	2379173	69005	34.478	34.042		
38-SET2	Fed Starve 1	2419887	71639	33.779			
39-SET2	Fed Starve 2	2226936	70031	31.799			
40-SET2	Fed Starve 3	2242496	77436	28.959	31.512	-0.07	0.3250
41-SET2	Fed Fed Fed (Control) 1	2131784	72808	29.280			
42-SET2	Fed Fed Fed (Control) 2	2397378	75519	31.745			
43-SET2	Fed Fed Fed (Control) 3	2246120	70087	32.048	31.024		
44-SET2	Fed Starve Fed (Rescue) 1	2410790	74304	32.445			
45-SET2	Fed Starve Fed (Rescue) 2	2178613	77108	28.254			
46-SET2	Fed Starve Fed (Rescue) 3	2327149	71001	32.776	31.158	0.00	0.9416

4725 **SD**
72484 **Average**
7% **Relative SD**

Component Name

mcm5s2U (5-methoxycarbonylmethyl-2-thiouridine)

Filename	Sample Type	Area	ISTD Area	Area Ratio	Average Ratio	Fold Change	p-value
32-SET2	Heat 1	2808974	60304	46.580			
33-SET2	Heat 2	2929213	64504	45.411			
34-SET2	Heat 3	2893043	60457	47.853	46.615	-0.06	0.0497
35-SET2	Fed Fed (Control) 1	3079787	60865	50.600			
36-SET2	Fed Fed (Control) 2	3179351	66203	48.025			
37-SET2	Fed Fed (Control) 3	3100005	61902	50.079	49.568		
38-SET2	Fed Starve 1	2696362	67745	39.802			
39-SET2	Fed Starve 2	2750050	61390	44.796			
40-SET2	Fed Starve 3	2777620	69378	40.036	41.545	-0.16	0.0230
41-SET2	Fed Fed Fed (Control) 1	3054864	70397	43.395			
42-SET2	Fed Fed Fed (Control) 2	3116881	65808	47.363			
43-SET2	Fed Fed Fed (Control) 3	3033944	62276	48.718	46.492		
44-SET2	Fed Starve Fed (Rescue) 1	3197326	67294	47.513			
45-SET2	Fed Starve Fed (Rescue) 2	2962085	63501	46.646			
46-SET2	Fed Starve Fed (Rescue) 3	2931855	59613	49.182	47.780	0.03	0.5204

3512 **SD**
64109 **Average**
6% **Relative SD**

Component Name

ms2t6A (2-methylthio-N6-threonyl carbamoyladenosine)

Filename	Sample Type	Area	ISTD Area	Area Ratio	Average Ratio	Fold Change	p-value
32-SET2	Heat 1	1985366	42096	47.163			
33-SET2	Heat 2	1780254	41185	43.226			
34-SET2	Heat 3	1881913	38700	48.629	46.339	0.00	0.9916
35-SET2	Fed Fed (Control) 1	1730540	42866	40.371			
36-SET2	Fed Fed (Control) 2	1894608	43790	43.266			
37-SET2	Fed Fed (Control) 3	2213584	39847	55.552	46.396		
38-SET2	Fed Starve 1	1620683	33081	48.991			
39-SET2	Fed Starve 2	2983670	42621	70.005			
40-SET2	Fed Starve 3	2275447	38622	58.916	59.304	0.28	0.1715
41-SET2	Fed Fed Fed (Control) 1	2511421	40087	62.649			
42-SET2	Fed Fed Fed (Control) 2	2574150	41801	61.581			
43-SET2	Fed Fed Fed (Control) 3	1974517	39041	50.576	58.269		
44-SET2	Fed Starve Fed (Rescue) 1	2214168	43157	51.305			
45-SET2	Fed Starve Fed (Rescue) 2	2680800	43557	61.547			
46-SET2	Fed Starve Fed (Rescue) 3	2163141	46641	46.379	53.077	-0.09	0.4298

3141 **SD**
41139 **Average**
8% **Relative SD**

Component Name

mG (methylated guanosine)

Filename	Sample Type	Area	ISTD Area	Area Ratio	Average Ratio	Fold Change	p-value
32-SET3	Heat 1	108054394	1884793	57.330			
33-SET3	Heat 2	110127463	1739487	63.310			
34-SET3	Heat 3	103667871	1846955	56.129	58.923	0.03	0.5506
35-SET3	Fed Fed (Control) 1	106246575	1867177	56.902			
36-SET3	Fed Fed (Control) 2	108356060	1837636	58.965			
37-SET3	Fed Fed (Control) 3	107119149	1912322	56.015	57.294		
38-SET3	Fed Starve 1	119153711	1977478	60.255			
39-SET3	Fed Starve 2	117464997	1814997	64.719			
40-SET3	Fed Starve 3	117256259	1985758	59.049	61.341	0.07	0.1285
41-SET3	Fed Fed Fed (Control) 1	123078793	1829034	67.292			
42-SET3	Fed Fed Fed (Control) 2	116536975	1881910	61.925			
43-SET3	Fed Fed Fed (Control) 3	118476504	1800836	65.790	65.002		
44-SET3	Fed Starve Fed (Rescue) 1	124586185	2017588	61.750			
45-SET3	Fed Starve Fed (Rescue) 2	123162807	1865581	66.018			

Filename	Sample Type	Area	ISTD Area	Area Ratio	Average Ratio	Fold Change	p-value
SMALL RNA FRACTION							
46-SET3	Fed Starve Fed (Rescue) 3	99046441	1823441	54.318	60.696	-0.07	0.3410

75166 **SD**
1872333 **Average**
4% **Relative SD**

Component Name
mG (methylated guanosine)

32-SET3	Heat 1	99062567	1677668	59.048			
33-SET3	Heat 2	95858498	1823826	52.559			
34-SET3	Heat 3	83081401	1567344	53.008	54.872	0.03	0.7114
35-SET3	Fed Fed (Control) 1	84647633	1667061	50.777			
36-SET3	Fed Fed (Control) 2	94233976	1919570	49.091			
37-SET3	Fed Fed (Control) 3	97846063	1632189	59.948	53.272		
38-SET3	Fed Starve 1	102207804	1851787	55.194			
39-SET3	Fed Starve 2	90931996	1891408	48.076			
40-SET3	Fed Starve 3	85681795	1751614	48.916	50.729	-0.05	0.5691
41-SET3	Fed Fed Fed (Control) 1	91648795	1932566	47.423			
42-SET3	Fed Fed Fed (Control) 2	81302966	1660698	48.957			
43-SET3	Fed Fed Fed (Control) 3	85586312	1630057	52.505	49.629		
44-SET3	Fed Starve Fed (Rescue) 1	101796506	1975492	51.530			
45-SET3	Fed Starve Fed (Rescue) 2	83036567	1649061	50.354			
46-SET3	Fed Starve Fed (Rescue) 3	76545888	1649261	46.412	49.432	0.00	0.9318

133827 **SD**
1751973 **Average**
8% **Relative SD**

Component Name
mG (methylated guanosine)

32-SET3	Heat 1	175323045	3378477	51.894			
33-SET3	Heat 2	183761158	3244959	56.630			
34-SET3	Heat 3	174270855	3161342	55.126	54.550	0.03	0.3473
35-SET3	Fed Fed (Control) 1	176327023	3278331	53.786			
36-SET3	Fed Fed (Control) 2	177916337	3470358	51.267			
37-SET3	Fed Fed (Control) 3	171653493	3220023	53.308	52.787		
38-SET3	Fed Starve 1	183066262	3611878	50.685			
39-SET3	Fed Starve 2	182233974	3470158	52.515			
40-SET3	Fed Starve 3	173676960	3325130	52.232	51.810	-0.02	0.3707
41-SET3	Fed Fed Fed (Control) 1	181738207	3453533	52.624			
42-SET3	Fed Fed Fed (Control) 2	173227888	3255344	53.213			
43-SET3	Fed Fed Fed (Control) 3	181332614	3189263	56.857	54.231		
44-SET3	Fed Starve Fed (Rescue) 1	195420528	3505888	55.741			
45-SET3	Fed Starve Fed (Rescue) 2	178426386	3216513	55.472			
46-SET3	Fed Starve Fed (Rescue) 3	161696576	3217196	50.260	53.824	-0.01	0.8642

138523 **SD**
3333226 **Average**
4% **Relative SD**

Component Name
m3G (N2,N2,7-trimethylguanosine)

32-SET3	Heat 1	3177518	23885	133.032			
33-SET3	Heat 2	2418684	26279	92.040			
34-SET3	Heat 3	3137492	22311	140.627	121.900	0.11	0.5004
35-SET3	Fed Fed (Control) 1	2820645	27972	100.839			
36-SET3	Fed Fed (Control) 2	2730778	23216	117.623			
37-SET3	Fed Fed (Control) 3	2803116	25546	109.730	109.397		
38-SET3	Fed Starve 1	3099118	24785	125.041			
39-SET3	Fed Starve 2	2790687	21345	130.740			
40-SET3	Fed Starve 3	3092408	26353	117.344	124.375	0.14	0.0765
41-SET3	Fed Fed Fed (Control) 1	2229765	19847	112.347			
42-SET3	Fed Fed Fed (Control) 2	2157507	22719	94.964			
43-SET3	Fed Fed Fed (Control) 3	1886641	24976	75.539	94.283		
44-SET3	Fed Starve Fed (Rescue) 1	2519163	27022	93.225			
45-SET3	Fed Starve Fed (Rescue) 2	2051075	20543	99.842			
46-SET3	Fed Starve Fed (Rescue) 3	3347631	21157	158.227	117.098	0.24	0.3986

2518 **SD**
23864 **Average**
11% **Relative SD**

Component Name
m22G (N2,N2-dimethylguanosine)

32-SET3	Heat 1	88448220	2188716	40.411			
33-SET3	Heat 2	107470385	2263188	47.486			
34-SET3	Heat 3	100417984	2321398	43.258	43.718	0.06	0.3624
35-SET3	Fed Fed (Control) 1	87365212	2116334	41.281			
36-SET3	Fed Fed (Control) 2	89678515	2106966	42.563			
37-SET3	Fed Fed (Control) 3	93863663	2343717	40.049	41.298		
38-SET3	Fed Starve 1	94480197	2273934	41.549			
39-SET3	Fed Starve 2	103200531	2190898	47.104			
40-SET3	Fed Starve 3	98131495	2304307	42.586	43.747	0.06	0.2872
41-SET3	Fed Fed Fed (Control) 1	102771339	2197307	46.771			
42-SET3	Fed Fed Fed (Control) 2	105309652	2273826	46.314			
43-SET3	Fed Fed Fed (Control) 3	102921208	2180180	47.208	46.764		
44-SET3	Fed Starve Fed (Rescue) 1	110507401	2384341	46.347			
45-SET3	Fed Starve Fed (Rescue) 2	109511204	2244021	48.801			
46-SET3	Fed Starve Fed (Rescue) 3	84864186	2148231	39.504	44.884	-0.04	0.5693

83150 **SD**
2235824 **Average**
4% **Relative SD**

Component Name
m1A (1-methyladenosine)

32-SET4	Heat 1	61004131	999328	61.045			
33-SET4	Heat 2	38427018	656415	58.541			
34-SET4	Heat 3	49705037	844436	58.862	59.483	0.04	0.4252
35-SET4	Fed Fed (Control) 1	51943580	839586	61.868			
36-SET4	Fed Fed (Control) 2	29545851	545373	54.176			
37-SET4	Fed Fed (Control) 3	47956351	870300	55.103	57.049		
38-SET4	Fed Starve 1	68294451	1058186	64.539			
39-SET4	Fed Starve 2	75897650	1056838	71.816			
40-SET4	Fed Starve 3	59507888	894848	66.501	67.618	0.19	0.0320

Filename	Sample Type	Area	ISTD Area	Area Ratio	Average Ratio	Fold Change	p-value
SMALL RNA FRACTION							
41-SET4	Fed Fed Fed (Control) 1	75277736	1092970	68.874			
42-SET4	Fed Fed Fed (Control) 2	60528114	922030	65.647			
43-SET4	Fed Fed Fed (Control) 3	63988631	945974	67.643	67.388		
44-SET4	Fed Starve Fed (Rescue) 1	73799504	1023103	72.133			
45-SET4	Fed Starve Fed (Rescue) 2	79419270	1101568	72.097			
46-SET4	Fed Starve Fed (Rescue) 3	53834708	971230	55.429	66.553	-0.01	0.8952
			157208	SD			
			921479	Average			
			17%	Relative SD			

Component Name
m6A (N6-methyladenosine)

32-SET4	Heat 1	13564162	305076	44.462			
33-SET4	Heat 2	14909374	288011	51.767			
34-SET4	Heat 3	13697042	293651	46.644	47.624	0.01	0.8553
35-SET4	Fed Fed (Control) 1	13957652	287958	48.471			
36-SET4	Fed Fed (Control) 2	14177958	310316	45.689			
37-SET4	Fed Fed (Control) 3	13692563	289389	47.316	47.158		
38-SET4	Fed Starve 1	13840697	334127	41.424			
39-SET4	Fed Starve 2	14265370	284611	50.122			
40-SET4	Fed Starve 3	13142753	259064	50.732	47.426	0.01	0.9385
41-SET4	Fed Fed Fed (Control) 1	13545808	307251	44.087			
42-SET4	Fed Fed Fed (Control) 2	14249078	307624	46.320			
43-SET4	Fed Fed Fed (Control) 3	13848275	313516	44.171	44.859		
44-SET4	Fed Starve Fed (Rescue) 1	15741257	323322	48.686			
45-SET4	Fed Starve Fed (Rescue) 2	15626717	329532	47.421			
46-SET4	Fed Starve Fed (Rescue) 3	13098877	270614	48.404	48.170	0.07	0.0274
			21083	SD			
			300271	Average			
			7%	Relative SD			

Component Name
t6A (N6-threonylcarbamoyladenosine)

32-SET4	Heat 1	17884303	354261	50.483			
33-SET4	Heat 2	24041222	389653	61.699			
34-SET4	Heat 3	19969970	386145	51.716	54.633	0.05	0.5699
35-SET4	Fed Fed (Control) 1	21733252	397914	54.618			
36-SET4	Fed Fed (Control) 2	22752825	444384	51.201			
37-SET4	Fed Fed (Control) 3	19633351	386995	50.733	52.184		
38-SET4	Fed Starve 1	19356085	374566	51.676			
39-SET4	Fed Starve 2	20266796	420434	48.204			
40-SET4	Fed Starve 3	18348244	358278	51.212	50.364	-0.03	0.3297
41-SET4	Fed Fed Fed (Control) 1	21687168	413612	52.434			
42-SET4	Fed Fed Fed (Control) 2	20066911	386651	51.899			
43-SET4	Fed Fed Fed (Control) 3	21703931	424086	51.178	51.837		
44-SET4	Fed Starve Fed (Rescue) 1	21051192	447530	47.039			
45-SET4	Fed Starve Fed (Rescue) 2	19802280	395258	50.100			
46-SET4	Fed Starve Fed (Rescue) 3	18974920	417050	45.498	47.545	-0.08	0.0778
			27845	SD			
			399788	Average			
			7%	Relative SD			

Component Name
i6A (N6-isopentenyladenosine)

32-SET4	Heat 1	5622033	475674	11.819			
33-SET4	Heat 2	2591834	493268	5.254			
34-SET4	Heat 3	3031881	455056	6.663	7.912	-0.13	0.6127
35-SET4	Fed Fed (Control) 1	5053266	474224	10.656			
36-SET4	Fed Fed (Control) 2	4301111	546195	7.875			
37-SET4	Fed Fed (Control) 3	4253412	478034	8.898	9.143		
38-SET4	Fed Starve 1	4140139	515052	8.038			
39-SET4	Fed Starve 2	5753852	488065	11.789			
40-SET4	Fed Starve 3	5563709	426052	13.059	10.962	0.20	0.3643
41-SET4	Fed Fed Fed (Control) 1	7239782	497945	14.539			
42-SET4	Fed Fed Fed (Control) 2	4594419	537017	8.555			
43-SET4	Fed Fed Fed (Control) 3	4128001	574547	7.185	10.093		
44-SET4	Fed Starve Fed (Rescue) 1	4422589	514657	8.593			
45-SET4	Fed Starve Fed (Rescue) 2	8066893	459953	17.539			
46-SET4	Fed Starve Fed (Rescue) 3	4235339	484338	8.745	11.625	0.15	0.7029
			38121	SD			
			494672	Average			
			8%	Relative SD			

Filename	Sample Name	Area	ISTD Area	Area Ratio	Average Ratio	Fold Change	P values
**LARGE RNA FRACTION**							

Component Name **Curve Index**
mC (methylated cytidine)

9-set1	Heat 1	NF	84267	NF			
10-set1	Heat 2	1538417	77244	19.916			
11-set1	Heat 3	1293830	96963	13.344	16.630	1.12	0.674986598
12-set1	Fed Fed (Control) 1	1019800	78388	13.010			
13-set1	Fed Fed (Control) 2	1459497	90760	16.081			
14-set1	Fed Fed (Control) 3	1471591	96304	15.281	14.790		
15-set1	Fed Starve 1	1796893	87700	20.489			
16-set1	Fed Starve 2	1740385	82703	21.044			
17-set1	Fed Starve 3	1958476	98852	19.812	20.448	1.38	0.015588956
18-set1	Fed Fed Fed (Control) 1	1704437	80618	21.142			
19-set1	Fed Fed Fed (Control) 2	1792428	91824	19.520			
20-set1	Fed Fed Fed (Control) 3	2218950	94603	23.455	21.373		
21-set1	Fed Starve Fed (Rescue) 1	1759376	100178	17.563			
22-set1	Fed Starve Fed (Rescue) 2	2103050	95266	22.076			
23-set1	Fed Starve Fed (Rescue) 3	1190678	99915	11.917	17.185	0.80	0.288979316
			8001	SD			
			90372	Average			
			9%	Relative SD			

Component Name
m1A (1-methyladenosine)

Filename	Sample Type	Area	ISTD Area	Area Ratio	Average Ratio	Fold Change	p-value
SMALL RNA FRACTION							
9-set1	Heat 1	17695713	784677	22.552			
10-set1	Heat 2	21537123	778096	27.679			
11-set1	Heat 3	19528316	802097	24.347	24.859	0.97	0.719827181
12-set1	Fed Fed (Control) 1	18156322	766355	23.692			
13-set1	Fed Fed (Control) 2	20551565	750365	27.389			
14-set1	Fed Fed (Control) 3	18824626	734134	25.642	25.574		
15-set1	Fed Starve 1	23459784	743028	31.573			
16-set1	Fed Starve 2	22659105	752249	30.122			
17-set1	Fed Starve 3	22913168	731060	31.342	31.012	1.21	0.023246069
18-set1	Fed Fed Fed (Control) 1	22348837	721271	30.985			
19-set1	Fed Fed Fed (Control) 2	22250689	766540	29.027			
20-set1	Fed Fed Fed (Control) 3	25137986	681744	36.873	32.295		
21-set1	Fed Starve Fed (Rescue) 1	21897033	768969	28.476			
22-set1	Fed Starve Fed (Rescue) 2	26236519	777920	33.727			
23-set1	Fed Starve Fed (Rescue) 3	17946351	787331	22.794	28.332	0.88	0.375621865

30841	**SD**
756389	**Average**
4%	**Relative SD**

Component Name
m5C (5-methylcytdine)

9-set1	Heat 1	19524832	1593775	12.251			
10-set1	Heat 2	24455358	1712566	14.280			
11-set1	Heat 3	21633584	1668176	12.968	13.166	1.04	0.571524316
12-set1	Fed Fed (Control) 1	19214735	1599297	12.014			
13-set1	Fed Fed (Control) 2	22080962	1646444	13.411			
14-set1	Fed Fed (Control) 3	20496996	1609897	12.732	12.719		
15-set1	Fed Starve 1	25099500	1663853	15.085			
16-set1	Fed Starve 2	24798635	1672118	14.831			
17-set1	Fed Starve 3	26673019	1727564	15.440	15.119	1.19	0.015302741
18-set1	Fed Fed Fed (Control) 1	24548690	1627215	15.086			
19-set1	Fed Fed Fed (Control) 2	24778039	1702245	14.556			
20-set1	Fed Fed Fed (Control) 3	26482320	1639832	16.149	15.264		
21-set1	Fed Starve Fed (Rescue) 1	24265232	1706990	14.215			
22-set1	Fed Starve Fed (Rescue) 2	26692007	1684527	15.845			
23-set1	Fed Starve Fed (Rescue) 3	19562856	1660266	11.783	13.948	0.91	0.386217211

41733	**SD**
1660984	**Average**
3%	**Relative SD**

Component Name
m7G (N7-methylguanosine)

9-set1	Heat 1	13875402	757578	18.315			
10-set1	Heat 2	17226825	778390	22.131			
11-set1	Heat 3	15449238	815858	18.936	19.794	0.99	0.854286378
12-set1	Fed Fed (Control) 1	14829661	813594	18.227			
13-set1	Fed Fed (Control) 2	15793256	742502	21.270			
14-set1	Fed Fed (Control) 3	15335517	738118	20.777	20.091		
15-set1	Fed Starve 1	19547416	745903	26.206			
16-set1	Fed Starve 2	17987299	743385	24.196			
17-set1	Fed Starve 3	19148419	798498	23.981	24.794	1.23	0.018847016
18-set1	Fed Fed Fed (Control) 1	20034923	745211	26.885			
19-set1	Fed Fed Fed (Control) 2	20773034	770786	26.950			
20-set1	Fed Fed Fed (Control) 3	23234817	701800	33.107	28.981		
21-set1	Fed Starve Fed (Rescue) 1	18165165	760406	23.889			
22-set1	Fed Starve Fed (Rescue) 2	24588504	802780	30.629			
23-set1	Fed Starve Fed (Rescue) 3	14309220	869741	16.452	23.657	0.82	0.330694501

41750	**SD**
772303	**Average**
5%	**Relative SD**

Component Name
Cm (2'-O-methyl-cytidine)

9-set1	Heat 1	68579622	4939344	13.884			
10-set1	Heat 2	74219324	5472524	13.562			
11-set1	Heat 3	76105965	5480052	13.888	13.778	0.98	0.387682034
12-set1	Fed Fed (Control) 1	73285019	5410499	13.545			
13-set1	Fed Fed (Control) 2	76982314	5397076	14.264			
14-set1	Fed Fed (Control) 3	78229510	5420250	14.433	14.080		
15-set1	Fed Starve 1	76785140	5403332	14.211			
16-set1	Fed Starve 2	75834899	5475234	13.851			
17-set1	Fed Starve 3	78459968	5776746	13.582	13.881	0.99	0.579949727
18-set1	Fed Fed Fed (Control) 1	73894413	5524067	13.377			
19-set1	Fed Fed Fed (Control) 2	75154563	5483917	13.705			
20-set1	Fed Fed Fed (Control) 3	79009309	5539709	14.262	13.781		
21-set1	Fed Starve Fed (Rescue) 1	74236181	5645257	13.150			
22-set1	Fed Starve Fed (Rescue) 2	78984705	5402391	14.620			
23-set1	Fed Starve Fed (Rescue) 3	76933011	5646987	13.624	13.798	1.00	0.975306592

182467	**SD**
5467826	**Average**
3%	**Relative SD**

Component Name
mG (methylated guanosine)

9-set1	Heat 1	8550233	517794	16.513			
10-set1	Heat 2	10587601	428993	24.680			
11-set1	Heat 3	8976997	434878	20.643	20.612	0.99	0.94150628
12-set1	Fed Fed (Control) 1	8675660	557272	15.568			
13-set1	Fed Fed (Control) 2	9839176	386723	25.442			
14-set1	Fed Fed (Control) 3	8295252	382294	21.699	20.903		
15-set1	Fed Starve 1	16212547	431825	37.544			
16-set1	Fed Starve 2	14396278	394078	36.532			
17-set1	Fed Starve 3	13743411	465668	29.513	34.530	1.65	0.024277359
18-set1	Fed Fed Fed (Control) 1	14470710	367470	39.379			
19-set1	Fed Fed Fed (Control) 2	14906427	559906	26.623			
20-set1	Fed Fed Fed (Control) 3	16121265	358640	44.951	36.984		
21-set1	Fed Starve Fed (Rescue) 1	13028236	419268	31.074			
22-set1	Fed Starve Fed (Rescue) 2	17908759	567361	31.565			

Filename	Sample Type	Area	ISTD Area	Area Ratio	Average Ratio	Fold Change	p-value
SMALL RNA FRACTION							
23-set1	Fed Starve Fed (Rescue) 3	7091935	611592	11.596	24.745	0.67	0.22686439

82781	**SD**
458918	**Average**
18%	**Relative SD**

Component Name
Gm (2'-O-methyl-guanosine)

9-set1	Heat 1	78577949	3184468	24.675			
10-set1	Heat 2	88961154	3312245	26.858			
11-set1	Heat 3	89083314	3351963	26.576	26.037	0.95	0.608695906
12-set1	Fed Fed (Control) 1	82636550	3563803	23.188			
13-set1	Fed Fed (Control) 2	87984582	2964437	29.680			
14-set1	Fed Fed (Control) 3	85215832	2932160	29.062	27.310		
15-set1	Fed Starve 1	87439272	2959339	29.547			
16-set1	Fed Starve 2	88818949	3014266	29.466			
17-set1	Fed Starve 3	84059205	3763954	22.333	27.115	0.99	0.953884521
18-set1	Fed Fed Fed (Control) 1	80223704	3187132	25.171			
19-set1	Fed Fed Fed (Control) 2	84374623	3449275	24.462			
20-set1	Fed Fed Fed (Control) 3	88628991	3113910	28.462	26.032		
21-set1	Fed Starve Fed (Rescue) 1	81854536	3201608	25.567			
22-set1	Fed Starve Fed (Rescue) 2	89656151	3593860	24.947			
23-set1	Fed Starve Fed (Rescue) 3	83973711	3853427	21.792	24.102	0.93	0.319509259

295008	**SD**
3296390	**Average**
9%	**Relative SD**

Component Name
m6A (N6-methyladenosine)

9-set1	Heat 1	10337295	366763	28.185			
10-set1	Heat 2	11186898	411305	27.199			
11-set1	Heat 3	10982064	406441	27.020	27.468	0.97	0.443049224
12-set1	Fed Fed (Control) 1	11462281	430056	26.653			
13-set1	Fed Fed (Control) 2	11144301	387858	28.733			
14-set1	Fed Fed (Control) 3	11320201	383689	29.504	28.297		
15-set1	Fed Starve 1	13660707	445574	30.659			
16-set1	Fed Starve 2	12111816	444788	27.231			
17-set1	Fed Starve 3	10855423	385801	28.137	28.676	1.01	0.790705632
18-set1	Fed Fed Fed (Control) 1	10953111	396567	27.620			
19-set1	Fed Fed Fed (Control) 2	13394460	485755	27.574			
20-set1	Fed Fed Fed (Control) 3	12499084	394849	31.655	28.950		
21-set1	Fed Starve Fed (Rescue) 1	11558162	417165	27.706			
22-set1	Fed Starve Fed (Rescue) 2	14700346	479345	30.668			
23-set1	Fed Starve Fed (Rescue) 3	11536061	396768	29.075	29.150	1.01	0.907721145

35110	**SD**
415515	**Average**
9%	**Relative SD**

Component Name
ac4C (N4-acetylcytidine)

9-set1_1511092023	Heat 1	863730	67289	12.836			
10-set2	Heat 2	1521352	79037	19.249			
11-set2	Heat 3	1135104	38458	29.515	20.533	0.87	0.67774791
12-set2	Fed Fed (Control) 1	932640	64786	14.396			
13-set2	Fed Fed (Control) 2	1242422	44874	27.687			
14-set2	Fed Fed (Control) 3	1107303	38896	28.468	23.517		
15-set2	Fed Starve 1	1750608	66393	26.367			
16-set2	Fed Starve 2	1708559	54440	31.384			
17-set2	Fed Starve 3	1689230	50767	33.274	30.342	1.29	0.272879049
18-set2	Fed Fed Fed (Control) 1	1832345	32321	56.692			
19-set2	Fed Fed Fed (Control) 2	1499492	54389	27.570			
20-set2	Fed Fed Fed (Control) 3	2139244		0.000	42.131		
21-set2	Fed Starve Fed (Rescue) 1	1653785	71203	23.226			
22-set2	Fed Starve Fed (Rescue) 2	2291916	46699	49.078			
23-set2	Fed Starve Fed (Rescue) 3	993809	40341	24.635	32.313	0.77	0.833132714

434891	14251	**SD**
1490769	53564	**Average**
29%	27%	**Relative SD**

Component Name
m62A (N6,N6-dimethyladenosine)

9-set1_1511092023	Heat 1	8243156	528633	15.593			
10-set2	Heat 2	12171548	647791	18.789			
11-set2	Heat 3	9899547	571669	17.317	17.233	0.90	0.213644782
12-set2	Fed Fed (Control) 1	10886417	610164	17.842			
13-set2	Fed Fed (Control) 2	12425786	664299	18.705			
14-set2	Fed Fed (Control) 3	12351752	590047	20.934	19.160		
15-set2	Fed Starve 1	13991695	641285	21.818			
16-set2	Fed Starve 2	12338611	703392	17.542			
17-set2	Fed Starve 3	12019742	617678	19.460	19.606	1.02	0.78774862
18-set2	Fed Fed Fed (Control) 1	13536748	581737	23.270			
19-set2	Fed Fed Fed (Control) 2	14477234	632058	22.905			
20-set2	Fed Fed Fed (Control) 3	16106719	664752	24.230	23.468		
21-set2	Fed Starve Fed (Rescue) 1	12873426	618872	20.801			
22-set2	Fed Starve Fed (Rescue) 2	17941988	667060	26.897			
23-set2	Fed Starve Fed (Rescue) 3	12895918	585456	22.027	23.242	0.99	0.915464126

2338501	45360	**SD**
12810686	621660	**Average**
18%	7%	**Relative SD**

Component Name
t6A (N6-threonylcarbamoyladenosine)

9-set1_1511092023	Heat 1	470466	15361	30.628			
10-set2	Heat 2	801014	16146	49.609			
11-set2	Heat 3	675623	25302	26.703	35.647	1.11	0.673011754

Filename	Sample Type	Area	ISTD Area	Area Ratio	Average Ratio	Fold Change	p-value
SMALL RNA FRACTION							
12-set2	Fed Fed (Control) 1	598380	17759	33.694			
13-set2	Fed Fed (Control) 2	729363	20697	35.240			
14-set2	Fed Fed (Control) 3	688037	25175	27.330	32.088		
15-set2	Fed Starve 1	1005732	18093	55.586			
16-set2	Fed Starve 2	1006066	20387	49.349			
17-set2	Fed Starve 3	1056509	14368	73.531	59.489	1.85	0.051572271
18-set2	Fed Fed Fed (Control) 1	834683	19554	42.687			
19-set2	Fed Fed Fed (Control) 2	914898	23554	38.843			
20-set2	Fed Fed Fed (Control) 3	1135708	18243	62.254	47.928		
21-set2	Fed Starve Fed (Rescue) 1	935316	19832	47.161			
22-set2	Fed Starve Fed (Rescue) 2	837742	16663	50.276			
23-set2	Fed Starve Fed (Rescue) 3	647238	22218	29.131	42.189	0.88	0.589747351

188118	3395	**SD**
822452	19557	**Average**
23%	18%	**Relative SD**

Component Name
i6A (N6-isopentenyladenosine)

9-set1_1511092023	Heat 1	48775	10540	4.628			
10-set2	Heat 2	93445	12817	7.291			
11-set2	Heat 3	51724	15819	3.270	5.063	0.76	0.350104533
12-set2	Fed Fed (Control) 1	64916	10004	6.489			
13-set2	Fed Fed (Control) 2	64855	12680	5.115			
14-set2	Fed Fed (Control) 3	124715	14760	8.449	6.684		
15-set2	Fed Starve 1	227232	10651	21.333			
16-set2	Fed Starve 2	61449	14647	4.195			
17-set2	Fed Starve 3	107466	11456	9.381	11.637	1.74	0.432969625
18-set2	Fed Fed Fed (Control) 1	170069	12379	13.738			
19-set2	Fed Fed Fed (Control) 2	112139	14907	7.522			
20-set2	Fed Fed Fed (Control) 3	261475	10246	25.519	15.593		
21-set2	Fed Starve Fed (Rescue) 1	167789	14812	11.328			
22-set2	Fed Starve Fed (Rescue) 2	292577	11557	25.316			
23-set2	Fed Starve Fed (Rescue) 3	115598	13942	8.291	14.979	0.96	0.938122679

77687	1959	**SD**
130948	12748	**Average**
59%	16%	**Relative SD**

Component Name
m5U (5-methyluridine)

9-set3	Heat 1	829699	72130	11.503			
10-set3	Heat 2	1261776	80129	15.747			
11-set3	Heat 3	1045959	79982	13.077	13.442	0.98	0.862422843
12-set3	Fed Fed (Control) 1	878837	70642	12.441			
13-set3	Fed Fed (Control) 2	1218859	75052	16.240			
14-set3	Fed Fed (Control) 3	1043188	82683	12.617	13.766		
15-set3	Fed Starve 1	1679223	82233	20.420			
16-set3	Fed Starve 2	1750403	79859	21.919			
17-set3	Fed Starve 3	1783681	84893	21.011	21.117	1.53	0.018205733
18-set3	Fed Fed Fed (Control) 1	1673737	78762	21.251			
19-set3	Fed Fed Fed (Control) 2	1519493	76877	19.765			
20-set3	Fed Fed Fed (Control) 3	1972552	82500	23.910	21.642		
21-set3	Fed Starve Fed (Rescue) 1	1559573	80734	19.318			
22-set3	Fed Starve Fed (Rescue) 2	1824141	86734	21.031			
23-set3-	Fed Starve Fed (Rescue) 3	875978	76997	11.377	17.242	0.80	0.275301762

4442	**SD**
79347	**Average**
6%	**Relative SD**

Component Name
Um (2'-O-methyl-uridine)

9-set3	Heat 1	5409900	352522	15.346			
10-set3	Heat 2	6593583	422927	15.590			
11-set3	Heat 3	6077633	411315	14.776	15.238	0.91	0.100230182
12-set3	Fed Fed (Control) 1	6125078	390118	15.701			
13-set3	Fed Fed (Control) 2	7015293	396443	17.696			
14-set3	Fed Fed (Control) 3	6217237	364932	17.037	16.811		
15-set3	Fed Starve 1	6457987	400956	16.106			
16-set3	Fed Starve 2	6909242	415992	16.609			
17-set3	Fed Starve 3	6590683	393791	16.737	16.484	0.98	0.64108887
18-set3	Fed Fed Fed (Control) 1	5676105	368665	15.396			
19-set3	Fed Fed Fed (Control) 2	6306333	384169	16.415			
20-set3	Fed Fed Fed (Control) 3	7166854	393660	18.206	16.673		
21-set3	Fed Starve Fed (Rescue) 1	5894245	375211	15.709			
22-set3	Fed Starve Fed (Rescue) 2	6556775	402589	16.287			
23-set3-	Fed Starve Fed (Rescue) 3	6594172	394670	16.708	16.235	0.97	0.656002548

19463	**SD**
391197	**Average**
5%	**Relative SD**

Component Name
m3G (N2,N2,7-trimethylguanosine)

9-set3	Heat 1	6233509	332338	18.757			
10-set3	Heat 2	8039450	387737	20.734			
11-set3	Heat 3	7675890	381793	20.105	19.865	0.84	0.008659064
12-set3	Fed Fed (Control) 1	8183764	354301	23.098			
13-set3	Fed Fed (Control) 2	8888360	371708	23.912			
14-set3	Fed Fed (Control) 3	8411230	347112	24.232	23.748		
15-set3	Fed Starve 1	9708612	351477	27.622			
16-set3	Fed Starve 2	10096361	385342	26.201			
17-set3	Fed Starve 3	10154247	371930	27.301	27.042	1.14	0.004564018
18-set3	Fed Fed Fed (Control) 1	9143427	343210	26.641			
19-set3	Fed Fed Fed (Control) 2	9390005	371516	25.275			
20-set3	Fed Fed Fed (Control) 3	10498221	396761	26.460	26.125		
21-set3	Fed Starve Fed (Rescue) 1	9751518	373311	26.122			
22-set3	Fed Starve Fed (Rescue) 2	11584498	395696	29.276			
23-set3-	Fed Starve Fed (Rescue) 3	9563479	348901	27.410	27.603	1.06	0.245049152

20159	**SD**

Filename	Sample Type	Area	ISTD Area	Area Ratio	Average Ratio	Fold Change	p-value
SMALL RNA FRACTION							
			367542	Average			
			6%	Relative SD			

Component Name
m22G (N2,N2-dimethylguanosine)

Filename	Sample Type	Area	ISTD Area	Area Ratio	Average Ratio	Fold Change	p-value
9-set3	Heat 1	3326221	173690	19.150			
10-set3	Heat 2	5681277	241224	23.552			
11-set3	Heat 3	4713425	225482	20.904	21.202	0.96	0.823561596
12-set3	Fed Fed (Control) 1	3803179	240202	15.833			
13-set3	Fed Fed (Control) 2	5242146	204948	25.578			
14-set3	Fed Fed (Control) 3	4437308	179841	24.674	22.028		
15-set3	Fed Starve 1	8440748	226633	37.244			
16-set3	Fed Starve 2	7689421	207363	37.082			
17-set3	Fed Starve 3	8460496	230262	36.743	37.023	1.68	0.040117466
18-set3	Fed Fed Fed (Control) 1	8310131	235113	35.345			
19-set3	Fed Fed Fed (Control) 2	7366132	261437	28.176			
20-set3	Fed Fed Fed (Control) 3	9869137	216984	45.483	36.335		
21-set3	Fed Starve Fed (Rescue) 1	7211759	233284	30.914			
22-set3	Fed Starve Fed (Rescue) 2	9233382	275319	33.537			
23-set3-	Fed Starve Fed (Rescue) 3	4339911	247115	17.562	27.338	0.75	0.27084787
			27329	SD			
			226593	Average			
			12%	Relative SD			
